# Wound Healing Metabolites from Peters’ Elephant-Nose Fish Oil: An In Vivo Investigation Supported by In Vitro and In Silico Studies

**DOI:** 10.3390/md19110605

**Published:** 2021-10-26

**Authors:** Faisal Alsenani, Ahmed M. Ashour, Mubarak A. Alzubaidi, Ahmed F. Azmy, Mona H. Hetta, Dalia H. Abu-Baih, Mahmoud A. Elrehany, Ahmed Zayed, Ahmed M. Sayed, Usama Ramadan Abdelmohsen, Abeer H. Elmaidomy

**Affiliations:** 1 Department of Pharmacognosy, Faculty of Pharmacy, Umm Al-Qura University, Makkah 21955, Saudi Arabia; fssenani@uqu.edu.sa; 2 Department of Pharmacology and Toxicology, Faculty of Pharmacy, Umm Al-Qura University, Makkah 21955, Saudi Arabia; amashour@uqu.edu.sa; 3 Department of Biological Sciences, Faculty of Science, King Abdulaziz University, Jeddah 21589, Saudi Arabia; mahalzubaidi@kau.edu.sa; 4 Department of Microbiology, Faculty of Pharmacy, Beni-Suef University, Beni Suef 62514, Egypt; ahmed.abdelaziz@pharm.bsu.edu.eg; 5 Department of Pharmacognosy, Faculty of Pharmacy, Fayoum University, Fayoum 63514, Egypt; mhm07@fayoum.edu.eg; 6 Department of Biochemistry and Molecular Biology, Faculty of Pharmacy, Deraya University, Minia 61111, Egypt; dalia.hamdy@deraya.edu.eg (D.H.A.-B.); mahmoud.elrehany@deraya.edu.eg (M.A.E.); 7 Department of Pharmacognosy, College of Pharmacy, Medical Campus, Tanta University, Elguish Street, Tanta 31527, Egypt; 8 Institute of Bioprocess Engineering, Technical University of Kaiserslautern, Gottlieb-Daimler-Str. 49, 67663 Kaiserslautern, Germany; 9 Department of Pharmacognosy, Faculty of Pharmacy, Nahda University, Beni Suef 62513, Egypt; ahmed.mohamed.sayed@nub.edu.eg; 10 Department of Pharmacognosy, Faculty of Pharmacy, Al-Maaqal University, Basra 61014, Iraq; 11 Department of Pharmacognosy, Faculty of Pharmacy, Minia University, Minia 61519, Egypt; 12 Department of Pharmacognosy, Faculty of Pharmacy, Deraya University, Minia 61111, Egypt; 13 Department of Pharmacognosy, Faculty of Pharmacy, Beni-Suef University, Beni Suef 62514, Egypt; abeerabdelhakium@yahoo.com

**Keywords:** Peters’ elephant-nose fish, GC-MS, fatty acids, COX, molecular modeling, molecular dynamic simulation, Inflammatory mediators, wound healing

## Abstract

*Gnathonemuspetersii* (F. Mormyridae) commonly known as Peters’ elephant-nose fish is a freshwater elephant fish native to West and Central African rivers. The present research aimed at metabolic profiling of its derived crude oil via GC-MS analysis. In addition, wound healing aptitude in adult male New Zealand Dutch strain albino rabbits along with isolated bioactive compounds in comparison with a commercial product (Mebo^®^). The molecular mechanism was studied through a number of in vitro investigations, i.e., radical scavenging and inhibition of COX enzymes, in addition to in silico molecular docking study. The results revealed a total of 35 identified (71.11%) compounds in the fish oil, belonging to fatty acids (59.57%), sterols (6.11%), and alkanes (5.43%). Phytochemical investigation of the crude oil afforded isolation of six compounds **1–6**. Moreover, the crude oil showed significant in vitro hydrogen peroxide and superoxide radical scavenging activities. Furthermore, the crude oil along with one of its major components (compound **4**) exhibited selective inhibitory activity towards COX-2 with IC_50_ values of 15.27 and 2.41 µM, respectively. Topical application of the crude oil on excision wounds showed a significant (*p* < 0.05) increase in the wound healing rate in comparison to the untreated and Mebo^®^-treated groups, where fish oil increased the TGF-β1 expression, down-regulated TNF-*α*, and IL-1β. Accordingly, Peters’ elephant-nose fish oil may be a potential alternative medication helping wound healing owing to its antioxidant and anti-inflammatory activities.

## 1. Introduction

Marine biodiversity has gained a special interest affording an enormous potential source of chemicals with many therapeutic and food applications [1]. Among them are fish, which are consumed worldwide owing to richness in protein, polyunsaturated fatty acids (PUFA or omega (ω)-fatty acids), and essential amino acids [2]. For instance, sardines, mackerel, anchovies, and some salmon species are the most important sources of polyunsaturated essential ω-3 fatty acids (ω-3 PUFA), including 20:5 ω-3 (eicosapentaenoic acid (EPA)) and 22:6 ω-3 (docosahexaenoic acid (DHA)). Such essential fatty acids show numerous nutraceutical benefits related to blood clotting [3], inflammatory response [4], functionality of the retina [5], central nervous system [6], and cardiovascular system [7,8].

Particularly, *Gnathonemus* *petersii*, F. Mormyridae (Peters’ elephant-nose fish) is a freshwater elephant fish, with other common names, i.e., elephant-nose fish, Ubangi mormyrid, and long-nosed elephant fish [9]. It is native to West and Central African rivers, specifically muddy, slow moving branches, and pools of the Niger, Chari, and Ogun Rivers [9]. It is a common Mormyrid in aquarium stores in the USA, while used as a food source for African people. Peters’ elephant-nose fish is characterized by a dark brown to black color with a lateral compressed body of an average length of 23–25 cm and a posterior dorsal and anal blade of an equal length [10]. The most peculiar feature is the trunk-like projection on the head suggesting its name. However, it is not a nose, it is an extension of the mouth used for several purposes including communication, self-defense against predators, navigation, and looking for its foods, i.e., worms and insects [10]. 

Wound healing of an excisional skin injury involves a cascade of complicated and dynamic mechanisms that represents a major clinical problem. Several approaches have been recommended warranting the vital structure and function of the injured tissue, including wound dressings and skin grafts transplantation [11]. In addition, antioxidants are usually recommended to regulate other medical consequences resulting from oxidative stress, i.e., damage of body tissue components as DNA, protein, and lipids [12]. However, synthetic drugs are commonly applied, which are accompanied with allergic reaction and drug resistance. So, naturally derived compounds are becoming potential candidates for wound healing [13].

Previous literature has revealed that unsaturated fatty acids (UFA) play potential roles modulating numerous factors involved in the wound healing process, including cell migration and proliferation, phagocytic capacity, and production of inflammatory markers [14]. Moreover, redox signaling facilitates normal tissue functions, as hemostasis, development and maturation of blood vessel formation and extracellular matrix, tissue formation, and wound closure [15].

Therefore, the present study investigated the chemical composition of Peters’ elephant-nose fish oil via gas chromatography-mass spectrometry (GC-MS) analysis and assessed its potential in the wound healing process exploring the most likely mechanisms of action, i.e., antioxidant and anti-inflammatory effects through in vitro and in silico studies. The results showed a rich metabolic profile of the produced oil, especially in fatty acids, and comparable wound healing aptitude to commonly used commercial products, i.e., Mebo^®^ and Celecoxib^®^.

## 2. Results 

### 2.1. GC-MS Analysis

Peters’ elephant-nose fish gave 1% v/w oil fresh weight, which is characterized by a yellow color, being odorless, lighter than water, and having a faint white turbidity at room temperature. The GC-MS analysis identified a total of 35 compounds representing 71.11% of the total detected peaks (Table 1, Figure S1). These identified compounds belonged to the following chemical classes: fatty acids, sterols, and alkanes. Fatty acids represented 59.57% of the total identified compounds followed by sterols (6.11%) and alkanes (5.43%), Table 1. Twenty-nine fatty acids (59.57%) were identified; varying from saturated fatty acids (14 SFA, 25.33%) representing the major fatty acids fraction, to mono-unsaturated fatty acids (5 MUFA, 12.16%), and poly unsaturated fatty acids (10 PUFA 22.08%), Table 1. The total unsaturated fatty acid (UFA) content was greater than that of the total SFA and accounted for 34.96%. Among UFA, palmitoleic and oleic acids were the most abundant MUFA, made-up of almost 3.4% and 4.99% of total MUFA, respectively. Combined n-3 PUFA (18:3, C20:3, C20:4, C32:4, C20:5, C18:2, and C22:6) accounted for 22.08% of total FA in crude Peters’ elephant-nose fish oil, which contained 4.63 and 5.55% of the long-chain fatty acids, i.e., 6,9,12,15-docosatetraenoic acid and 4,7,10,13,16,19-docosahexaenoic acid, respectively. Furthermore, sterol represented 6.11%, made-up mainly of cholesterol and its derivatives. In addition, higher alkane (5.43%) was represented by heptacosane and dotriacontane, Table 1.

### 2.2. Physicochemical Investigation of Oil

Physicochemical and chromatographic properties, the spectral analyses, i.e., UV, ^1^H, and DEPT-Q NMR, as well as comparisons with previous reports and some standards showed that the oil extract of Peters’ elephant-nose fish afforded the following known compounds: palmitoleic acid **1** [16], palmitic acid **2** [16]. Additionally, oleic acid **3** [17], 6,9,12,15-docosatetraenoic acid **4** [18], cholesterol **5** [19], and 2-decylphenol **6** [20], Figure 1 & Figures S2–S13.

### 2.3. In Vitro COX-1 and COX-2 Inhibitory Activity

Fatty acids, especially unsaturated ones, demonstrate great potential in promoting wound healing. In addition, the topical application of unsaturated fatty acids-rich oils was very effective in controlling inflammation associated with skin wounds [21,22,23]. The COX pathway plays a crucial role in the complex wound healing process, and its major product prostaglandin E2 (PGE-2) is among the main inflammatory mediators that are involved in the inflammatory phase of wound healing and its associated pain [23,24]. Recently, it has been reported that selective inhibition of COX-2 was associated with reduced inflammation and accelerated wound healing in experimental animals [25]. Consequently, depending on the structural similarity between the isolated fatty acids and arachidonic acid (AA, i.e., the main precursor of COX enzyme, their inhibitory activity against COX-1 and COX-2 were examined. The results showed that, the crude oil and only compound **4** were active against both enzymes with significant selectivity toward COX-2, the known Celecoxib was used as a positive control, Table 2. Hence, compound **4** could be one of the bioactive components in Peters’ elephant-nose fish oil that may contribute to its wound healing activity and anti-inflammatory potential.

### 2.4. Molecular Modeling

**Figure 2:**
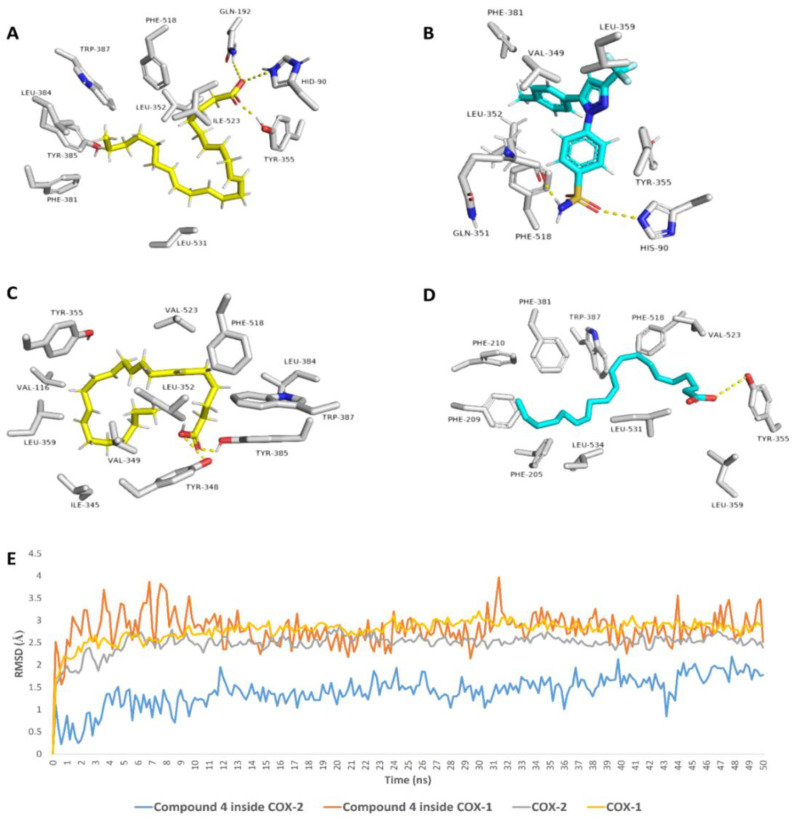
Binding modes of compound **4** inside the binding pockets of both COX-1 (**A**) and COX-2 (**C**) and its MDS (**E**). (**B**,**D**) are the binding modes of the co-crystalized ligands of each enzyme.

To have some insight into the binding modes of compound **4** with both COX1 and COX-2, we docked it against the crystal structures of both enzymes (PDP codes: 3KK6 and 3HS5, respectively). It achieved docking scores of −6.9 and −8.4 kcal/mol, respectively. Such a difference in docking scores may explain the difference in the inhibitory activity, Table 3. Inside the binding pocket of COX-1, compound **4**’s fatty tail was able to achieve multiple hydrophobic interactions with several hydrophobic amino acid’s side chains, e.g., LEY-352, PHE-381, LEU-384, TYR-385, TRP-387, PHE-518, ILE-523, and LEU-531, while the hydrophilic carboxylate moiety was able to form two H-bonds with GLN-192 and TYR-355 in addition to an ionic interaction with HID-90, Figure 2A,B. Similarly, inside the COX-2’s active site, Figure 2C, compound **4** was also extensively interacted with 9-hydrophobic residues through its hydrophobic long tail (e.g., VAL-116, ILE-345, VAL-349, LEU-352, TYR-355, LEU-359, LEU-384, TRP-387, and PHE-518). In contrast to the co-crystalized ligand which carboxylate moiety forms a single H-bonds with TYR-355, Figure 2D, compound **4**’s carboxylate moiety formed two H-bonds with two other tyrosine residues, i.e., TYR-348 and TYR-385 on the opposite side.

Further MDS-based in silico investigations were performed on the two docking poses of compound **4** with both COX-1 and COX-2 to validate its binding mode and to calculate its binding free energies (ΔGs) with both enzymes. As shown in Figure 2E, compound **4** was able to reach equilibrium rapidly (at 1.3 ns) inside COX-2’s binding pocket and its deviation from the starting docking pose was small (Average RMSD = 0.9 Å). This was not the same case inside COX-1’s binding pocket, where it strongly deviated and fluctuated at the beginning of the simulation, and then started to equilibrate at 2.9 ns around RMSD of 2.9 Å. This difference in stability inside the binding pocket of each enzyme was translated in different calculated ΔGs, which was comparable with their docking scores (ΔG = −5.6 and −8.1 kcal/mol), and hence, such differences in binding modes and stability may explain the preferential inhibitory activity of compound **4** toward COX-2.

### 2.5. In Vitro Antioxidant Activity

#### 2.5.1. Hydrogen Peroxide Scavenging 

**Figure 3:**
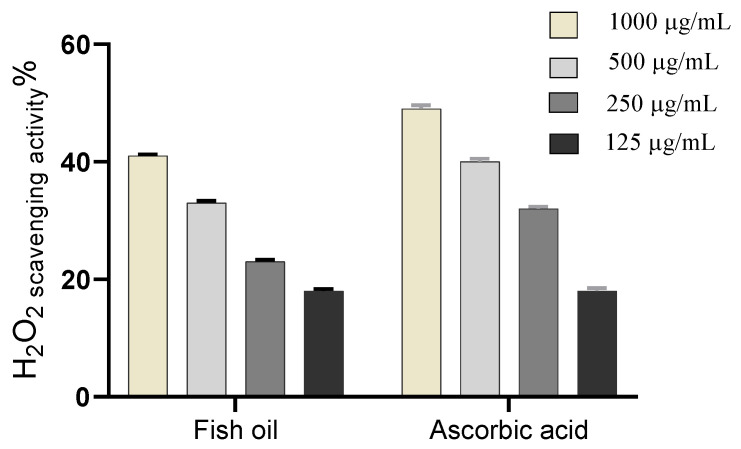
H_2_O_2_ radical scavenging activity of Peters’ elephant-nose fish oil.

In this study, Peters’ elephant-nose fish oil was evaluated for its antioxidant activity as scavenger potential against H_2_O_2_. The results showed that, maximum H_2_O_2_ radical scavenging activity for the oil was 41% at 1000 μg/mL for H_2_O_2_. Fish oil significantly inhibited the generation of H_2_O_2_ radicals in a dose-dependent manner reflecting a reliable antioxidant activity with IC_50_ of 171.1 μg/mL, Figure 3, compared to ascorbic acid (IC_50_ = 174.2 μg/mL) used as a positive control [12].

#### 2.5.2. Superoxide Radical Scavenging 

**Figure 4:**
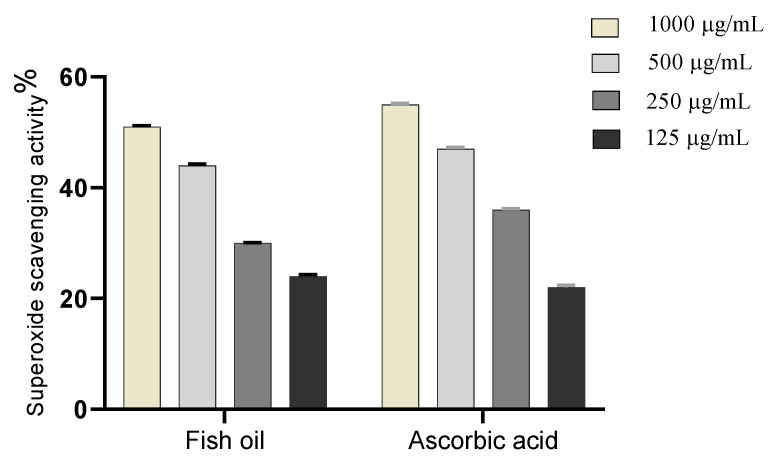
Superoxide radical scavenging activity of Peters’ elephant-nose fish oil.

Peters’ elephant-nose fish oil was evaluated for its superoxide scavenging activity and the results showed that, the crude oil significantly scavenged the superoxide radical in a dose-dependent manner reflecting a reliable antioxidant activity with IC_50_ of 153.7 µg/mL, Figure 4, compared to ascorbic acid (IC_50_ = 164 μg/mL) [15].

### 2.6. Wound Healing Activity

#### 2.6.1. Wound Closure Rate Estimation

**Figure 5:**
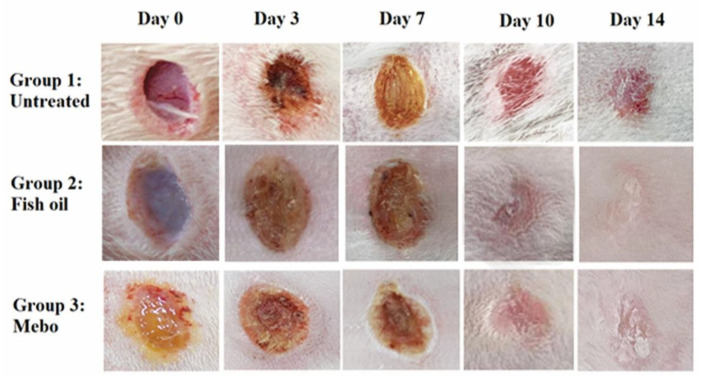
The excisional wound results on days 0, 3, 7, 10, and 14 post-wounding for Peters’ elephant-nose fish oil and Mebo^®^ in wound of adult male New Zealand Dutch strain albino rabbits, group 1: untreated (positive control), group 2: Peters’ elephant-nose fish oil treated group, and group 3: Mebo^®^-treated group.

In response to wound processes, the excisional wounds on days 0, 3, 7, 10, and 14 post-wounding were detected in all tested groups (group 1: untreated group positive control, group 2: Peters’ elephant-nose fish oil treated group, group 3: Mebo^®^-treated group), Figure 5. 

**Figure 6:**
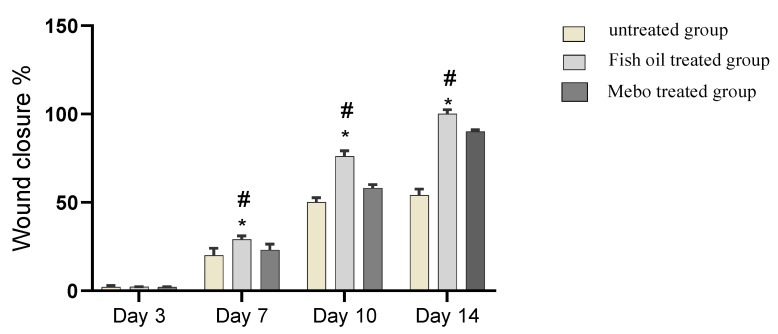
Wound closure rates in all tested groups (group 1: untreated group positive control, group 2: Peters’ elephant-nose fish oil treated group, group 3: Mebo^®^-treated group) over time post-injury. Data were expressed as mean ±S.E.M. * *p* < 0.05 compared with those of the untreated group on the respective day and # *p* < 0.05 compared with those of the Mebo^®^ group on the respective day.

Results showed that no apparent difference in wound closure rate was observed between all tested groups on the 3^rd^ day. On the 7^th^ day post-injury, the wound closure in the fish oil treated group was 29%, which appeared to be significantly higher (*p* < 0.05) than the corresponding untreated group. In addition, the fish oil treated group also showed improved wound closure rates in comparison to the Mebo^®^-treated group (23%) (*p* < 0.05). The wound closure rates of the fish oil treated group (76%) were again significantly higher (*p* < 0.05) than the untreated group (58%) on the 10th day post-injury. On 14th day post-injury, the wounds in fish oil treated group were completely healed and the wound closure reached 100% and 90% in fish oil treated group and Mebo^®^-treated group, respectively, Figure 6.

#### 2.6.2. Histopathological Study 

##### 7 Days after Treatment

Group 1 (untreated group)

**Figure 7:**
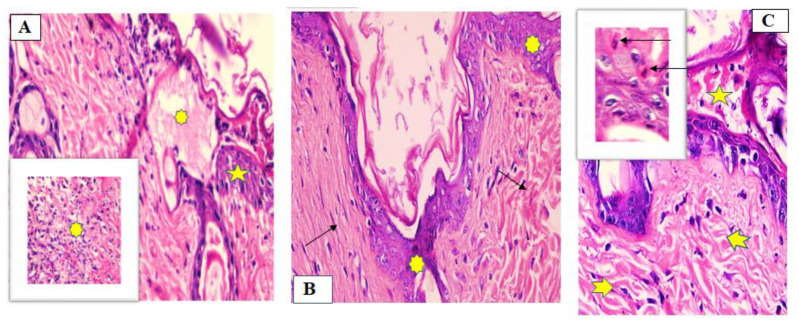
Wounded skin 7 days after incision and treatment showing (**A**) the normal edge of the wound with normal epidermis (star) and the wound is filled with blood clots, sloughed granulation tissue (asterisk) for Group I (untreated group), (**B**) granulation tissue filing the base of the defect is mainly fibrous. Collagen bundles as coarse and wavy bundles (green arrows) for Group II (fish oil-treated group), and (**C**) scare tissue blocking the wound (star), collagen fibers (arrow heads) resembling that of the adjacent normal dermis. Inflammatory cellular mainly of macrophages (black arrows) was also observed for Group III (Mebo^®^-treated group). (Hematoxylin and eosin stain ×200 and ×400).

The normal edge of the wound appeared with normal epidermis, well-formed dermal collagen bundles, normal hair follicles and sebaceous glands. On the other hand, the wound is filled with blood clots, sloughed granulation tissue with collagen fibers compactly arranged in an abnormal pattern, extravasated RBCs and inflammatory cellular infiltration. The striated muscle showed necrotic myofibers in the deepest part of the wound, Figure 7A.

Group II (fish oil-treated group)

There was complete re-epithelization covering the defect. The granulation tissue filling the base of the defect was mainly fibrous. The reticular dermis with collagen bundles as coarse and wavy bundles arranged in different directions, and many newly formed hair follicles, Figure 7B.

Group III (Mebo^®^-treated group)

Scare tissue blocking the wound and creeping of epidermal cells at the edges of the wound was observed but the re-epithelization was incomplete. Marked inflammatory cellular infiltration (mainly macrophages), and collagen fibers were seen filling the defect in a reticular pattern with spacing in between almost resembling that of the adjacent normal dermis. The reticular dermis containing frequent active elongated, spindle-shaped fibroblasts with basophilic cytoplasm and open face oval nuclei, Figure 7C.

##### 14 Days after Treatment

Group I (untreated group)

**Figure 8:**
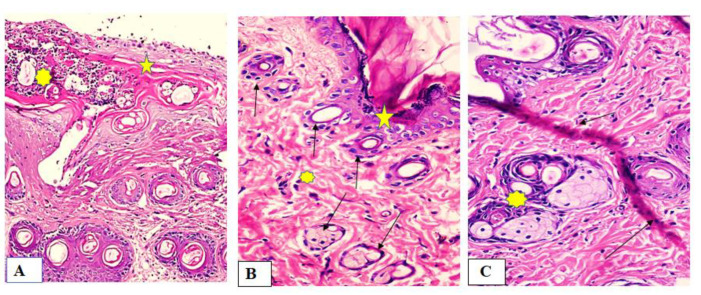
Wounded skin 14 days after incision and treatment showing (**A**) wide wound area (star) heavy inflammatory cellular infiltration in an acidophilic matrix (asterisk) and the normal skin (star) for group I (untreated group), (**B**) typical stratified squamous keratinized epithelium (star) and dermal matrix with coarse wavy collagen bundles in different directions (asterisk). Notice numerous newly formed hair follicles (green arrows) for Group II (fish oil treated group), and (**C**) typical epithelium, thin scar tissue extending into the dermis (black arrows), reticular dermis has coarse wavy collagen bundles arranged in different directions. Newly formed hair follicles (asterisk) for Group III (Mebo^®^-treated group). (Hematoxylin and eosin stain ×200 and 400).

The wound area appeared wider and filled with a thick layer of granulation tissue which is formed of several layers of connective tissue cells in an acidophilic matrix and overlying heavy inflammatory cellular infiltration. The dermis is formed of disorganized, thin collagen with marked neovascularization, Figure 8A.

Group II (fish oil-treated group)

Both epidermis and dermis appeared normal. There was complete re-epithelization covering the defect (typical stratified squamous keratinized epithelium). The dermal matrix with numerous hair follicles, numerous blood capillaries and the collagen bundles in the papillary layer appeared as fine interlacing bundles, and in the reticular layer appeared as coarse wavy bundles, Figure 8B.

Group III (Mebo^®^-treated group)

The skin tissue appeared normal with typical stratified squamous keratinized epithelium. Thin scare tissue may extend into the dermis. The dermal matrix with many hair follicles, blood capillaries and absence of inflammatory cellular infiltration. The collagen bundles in the papillary dermis appeared as fine interlacing bundles, while as coarse wavy bundles arranged in different directions in the reticular dermis, Figure 8C.

#### 2.6.3. Effect of Fish Oil on mRNA Expression of TGF-β in Experimental Animals

**Figure 9:**
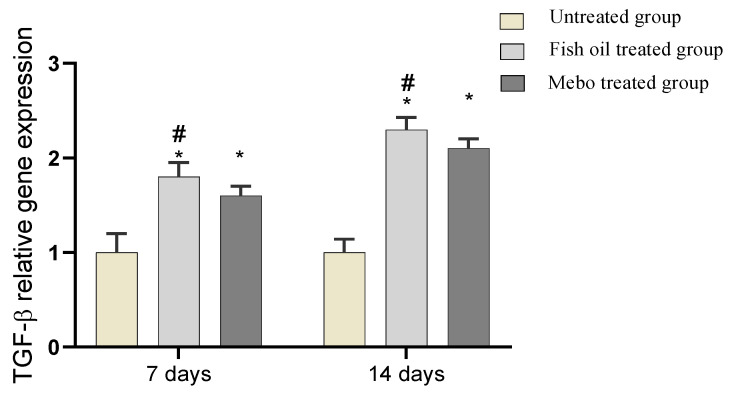
Gene expression in wound tissues for rabbits of different groups. Bars represent mean ± SD. Significant difference between groups was analyzed by a one-way ANOVA test, where: * *p* < 0.05 compared with the untreated group on the respective day and # *p* < 0.05 compared with the Mebo^®^ group on the respective day. Data represent fold change in relation to the normal control group after normalization to glyceraldehyde 3-phosphate dehydrogenase (GAPDH).

The tissues’ relative mRNA expression of TGF-β was significantly increased in fish oil treated wounds for 7 or 14 days comparing to the untreated group (*p* < 0.05). Moreover, the relative expression showed significant increase in the TGF-*β* expression in comparison to the Mebo^®^-treated group, Figure 9.

#### 2.6.4. In Vivo Effect of Fish Oil on mRNA Expression of Inflammatory Markers, i.e., TNF-α and IL-1β

**Figure 10:**
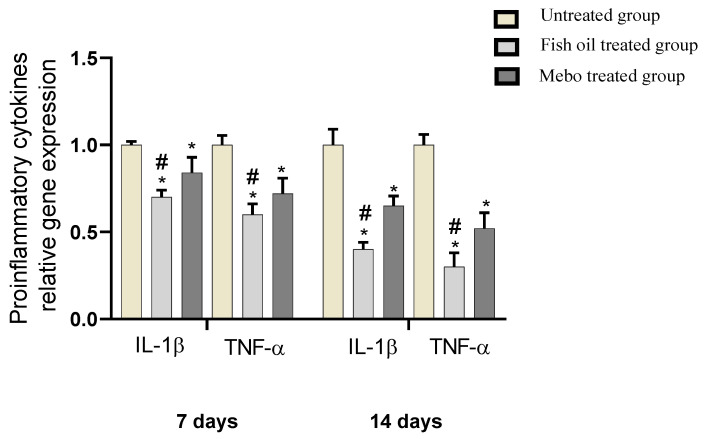
Gene expression in wound tissues for rabbits of different groups. Bars represent mean ± SD. Significant difference between groups was analyzed by a one-way ANOVA test, where: * *p* < 0.05 compared with the untreated group on the respective day and # *p* < 0.05 compared with the Mebo^®^-treated group on the respective day. Data represent fold change based on the normal control group expression after normalization to glyceraldehyde 3-phosphate dehydrogenase.

Analysis of mRNA expression of a full-thickness wound samples on 7 and 14 days post-injury revealed a downregulation of the inflammatory mediators activity, i.e., TNF-*α* and IL-1β, significantly in a time dependent manner in both of Group II and Group III, in comparison with Group I (*p* < 0.05). However wounded rabbits treated with fish oil (Group II) displayed a more significant reduction in TNF-α and IL-1β, compared to Mebo^®^-treated group (Group III) (*p* < 0.05), Figure 10.

## 3. Discussion

It has been proven that the wound healing process encompasses three phases, i.e., inflammatory, proliferative, and remodeling. The inflammatory phase occurs as a result of the production of pro-inflammatory mediators and suppression of the immune response. It is followed by a proliferative phase which occurs over the course of fibroblast proliferation, the accumulation of collagen, and angiogenesis. Finally, the remodeling phase involves the collagen remodeling and tissue reconstruction [26,27]. Treatments that expedite wound healing and may contribute at all stages of the wound healing process will be required for efficient treatment at a cheap cost and with fewer adverse effects.

Topical application of fish oil to wounded animals showed a significant (*p* < 0.05) reduction in wound area, in contrast to the untreated wounds, as illustrated in Figure 5. This result may be explained by the accelerated wound contraction rate in fish oil treated wounds, if the wound contraction is described as the centripetal migration of the full thickness edges of a wound allowing for wound closure [28,29,30]. Thus, wound contraction can be applied as an indicator of re-epithelialization, fibroblast proliferation, angiogenesis, granulation, and proliferation [30].

In addition, the pronounced increase in wound closure rate exhibited by fish oil was confirmed by histological studies. In all specimens, epidermal repair was at an advanced stage by the termination of the experiment at the fourteenth day (day 14), Figure 8. Application of Mebo^®^ induced partial improvement of the healing process of wounded animals. In contrast, fish oil treatment resulted in obvious acceleration of the healing process with improved re-epithelization, neovascularization, and reorganization of the dermis with significantly increased collagen deposition with prominent fibroblasts and new hair follicle formation. 

In the first seven days post wounding there was predominance of inflammatory phase in untreated group. While in fish oil or Mebo^®^-treated groups there was predominance of proliferative phase including decrease inflammation, reepithelization, collagen deposition and new hair follicle formation with the fish oil gives the best results of acceleration of wound healing, Figure 7.

It could be concluded that rapid re-epithelialization occurred in fish oil-treated animals, which is consistent with previous findings of Tse-Hung Huang et al., 2018 [31] who reported that the major mechanisms for fish oil application was attenuating cutaneous inflammation, and fish oil may act as the regulator affecting the production and activity of cytokines used for wound healing.

The healing processes encompass complicated interactions between cells as well as a variety of growth factors [32]. During the wound healing process, TGF-β is the most crucial player as in hemostasis and inflammatory phase TGF-β can activate and recruit various inflammatory cells including neutrophils and macrophages. On the other hand, TGF-β could manage numerous cellular events in the proliferative phase such as extracellular matrix deposition, granulation tissue formation, as well as re-epithelialization and angiogenesis [32]. Moreover, TGF-β stimulates fibroblast proliferation and differentiation into myofibroblasts which accelerate wound contraction [33]. It is also noteworthy to mention that previous reports have shown that chronic non-healing wounds often demonstrate a loss of TGF-β signaling, and hence, a decreased level of TGF-β [34,35]. In addition, Feinberg et al., demonstrated that TGF-β can down-regulate collagenase expression that acts on collagen and extracellular matrix [36]. Thus, the obtained results agreed with previous reports, where mRNA expression analysis of the wound tissues showed an increment in TGF-β1 levels in fish oil-treated wound tissues compared to the untreated wound tissues. This may suggest that fish oil improved the wound healing process via stimulating the expression of TGF-β1 in the wound tissues, Figure 9.

Furthermore, the pro-inflammatory cytokines, i.e., IL-1β, and TNF-α are required only in the inflammatory phase (the first phase of wound healing), and therefore, controlled IL-1β and TNF-α expression is necessary to attract neutrophils, Additionally, IL-1β and TNF-α are important for bacterial and contaminants elimination from the injury site, as well as stimulating the synthesis of Metalloproteinase (MMP). In the healing process, damaged extracellular matrixes (ECM) is degraded by MMPs in order to improve tissue restoration [37]. However, prolonged inflammation may result in a delayed wound healing as those released cytokines and proteinase may cause tissue destruction leading to chronic wounds. It has been reported that upregulated pro-inflammatory cytokines were associated with impaired wound healing. This explains the improved wound healing effect of fish oil via suppressing inflammatory cytokines TNF-α and IL-1β which in turn inhibits prolonged inflammation and hence avoid impaired wound healing. These results confirmed that fish oil justified the anti-inflammatory responses promoting the healing process, Figure 10.

As reported previously, inflammation is the early phase of wound healing process however, prolonged inflammation can impair the healing process and the wound may enter a pathological state which would require more intensive treatment. COX-2, the key inflammation regulator, is not detected in most normal tissues, but its expression is rapidly induced by stimuli such as proinflammatory cytokines (IL-1b, TNFα). The inducible COX-2 has been implicated in inflammation as it increases the synthesis of PGs and hence inflammation. The ability of an agent to resolve the inflammation will promote or accelerate the healing process [38]. In this context COX-1 and COX-2 inhibiting potential were evaluated and the crude fish oil together with compound **4** were found to exhibit preferential inhibitory activity against COX-2, and hence, it may contribute to their wound-healing activity, Table 2.

To have some insight into the binding modes of compounds 4 with both COX-1 and COX-2, we docked it against the crystal structures of both enzymes. Subsequently, its binding mode and stability were monitored over 50 ns of MDS. The structure of compound **4** was found to be more stable inside the active site of COX-2 and such stability was achieved via its multiple stable interactions, i.e., H-bonding and hydrophobic interactions, with the active site’s amino acid residues. Accordingly, compound **4**’s estimated ΔG value with COX-2 was significantly lower than that with COX-1 (Δ*G* = −8.1 and −5.6 kcal/mol, respectively), and hence, such differences in binding modes and stability may explain the preferential inhibitory activity of compound **4** toward COX-2.

On the other hand, high levels of reactive oxygen species (ROS), O_2_, OH, and H_2_O_2_ in wound site may promote collagen breakdown and ECM destruction resulting in inhibition of multiple processes which are crucial for wound healing such as angiogenesis and re-epithelization [39,40]. Furthermore, elevated ROS can elevate pro-inflammatory cytokines and hence prolong inflammation [41]. The current study revealed that fish oil has SOD and H_2_O_2_ scavenging activity that attribute to its antioxidant activity and hence enhance its wound-healing activity, Figure 3 and Figure 4.

## 4. Materials and Methods

### 4.1. Fish Collection

Peters’ elephant-nose fish were purchased during June 2020 from a local market, Beni-Suef, Egypt, then identified using fish identification key [42]. A voucher specimen (2020-BuPD 80) was archived at the Department of Pharmacognosy, Faculty of Pharmacy, Beni-Suef University, Egypt. After purchasing, the fish was stored in plastic bags and preserved using dry ice. 

### 4.2. Chemicals and Reagents

Methanol (MeOH), n-hexane (n-hex., boiling point b.p. 60–80 °C), dichloromethane (DCM), ethyl acetate (EtOAC), H_2_O_2_, sulphuric acid, and sodium bicarbonate were purchased from El-Nasr Company for Pharmaceuticals and Chemicals, Cairo, Egypt. However, deuterated solvents, including chloroform (CDCl_3_), and dimethyl sulfoxide (DMSO-d6) used for spectroscopic analyses were provided by Sigma-Aldrich (Saint Louis, MO, USA). 

Column chromatography (CC) was carried out using silica gel 60 (63–200 μm, E. Merck, Sigma-Aldrich), while thin-layer chromatography (TLC) silica gel G F_254_ (El-Nasr Company for Pharmaceuticals and Chemicals, Egypt) was employed for vacuum liquid chromatography (VLC). TLC was performed using pre-coated silica gel 60 GF254 plates (E. Merck, Darmstadt, Germany; 20 × 20 cm, 0.25 mm in thickness). Spots were visualized by heating at 110 °C, according to [43], after spraying with para-anisaldehyde (PAA) reagent (85:5:10:0.5 absolute EtOH: sulfuric acid: glacial acetic acid: para-anisaldehyde) [43]. 

In addition, all the kits used for the biological study were purchased by Biosystems SA Costa Brava 30, Barcelona (Spain) and DiaSys Diagnostic Systems GmbH, Holzheim, Germany.

### 4.3. NMR Spectral Analyses

Bruker Advance III 400 MHz with BBFO Smart Probe and a Bruker 400 MHz EON Nitrogen-Free Magnet (Bruker AG, Billerica, MA, USA) was used for proton ^1^H and Distortionless Enhancement by Polarization Transfer-Q (DEPT-Q) ^13^C NMR analyses. Spectra were recorded at 400 and 100 MHz for ^1^H and ^13^C measurement, respectively, where tetramethylsilane (TMS) was used as an internal standard in chloroform (CDCl_3_) and dimethyl sulfoxide (DMSO-d6). The residual solvent peaks (*δ*_H_ = 7.2) and (*δ*_H_ = 2.50 ppm and *δ*_C_ = 39.5 ppm) were recognized as references, respectively. Carbon multiplicities were determined using a DEPT-Q experiment. Finally, HRESI-MS analysis was performed with an Acquity Ultra Performance Liquid Chromatography system coupled to a Synapt G2 HDMS quadrupole time-of-flight hybrid mass spectrometer (Waters, Milford, MA, USA). 

### 4.4. Sample Preparation and Lipid Extraction 

The analyses of fish sampled were performed in accordance with the procedure in AOAC (2006). In the laboratory, the fish sample (1000 g) was thawed, beheaded, degutted, washed with water, followed by grinding using an OC-60B/60B grinding machine (60–120 mesh, Luohe, China). MeOH and DCM at a ratio of 2:1 (3L, 2×, three days each) were used for lipid extraction. Then, the extract was stored in a refrigerator, filtered, and dehydrated by adding sodium sulphate anhydrous. Finally, the extract was concentrated by solvents evaporation, at room temperature, for 24 h affording 75 g of a crude extract. 

Afterwards, the dried extract was re-suspended in 50 mL distilled water and successively portioned with solvents of different polarities (n-Hex., DCM, EtOAC, and n-butanol). The organic phase in each step was treated as before, and then evaporated under reduced pressure to produce the corresponding fractions; I (25.0 g), II (1.5 g), III (1.5 g) and IV (15.0 g), respectively, while the aqueous remaining mother liquor was also concentrated to yield fraction (V). All fractions were stored at 4 °C, until further biological and phytochemical investigations performed.

### 4.5. Preparation of Fatty Acids Methyl Esters 

Methylation was performed according to [44]. In brief, 5 mg of fraction I were suspended in 1 mL n-hexane. Then, a 2 mL aliquote of methanolic sulfuric acid (1%, *v*/*v*) was added in vials and sealed. The sample was heated in a stopper tube at a temperature of 50 °C for 16 h. To end up the reaction, 2 mL aqueous sodium bicarbonate (2%, *w*/*v*) were added. Then, hexane (2 × 5 m) was applied to extract products. Finally, samples were concentrated at room temperature to remove acids for 48 h.

### 4.6. GC-MS Analysis of Fatty Acids Methyl Esters

The recovered fatty acids methyl esters were subjected to chromatographic analysis using GC-MS [45]. The GC-MS instrument was a TRACE^®^ GC Ultra Gas Chromatograph (Thermo Scientific Corp., Berkeley, MO, USA), coupled with a Thermo MS detector (ISQ^®^ Single Quadrupole Mass Spectrometer, Thermo Fisher Scientific, Berkeley, MO, USA). The system was equipped with a TR-5 MS column (30 m × 0.32 mm i.d., 0.25 μm film thickness). 

The system was programmed to perform analysis using 1 μL diluted samples (1:10 hexane, *v/v*), helium as carrier gas, and the injector and detector were held at 210 °C. The flow ray was adjusted at a 1.0 mL/min and a split ratio of 1:10. The temperature program was 60 °C for 1 min; rising at 4.0 C/min to 240 °C and held for 1 min. Mass spectra were obtained by electron ionization (EI) at 70 eV, using a spectral range of *m/z* 40-450. Finally, the obtained MS data were de-convoluted using AMDIS software (www.amdis.net, accessed on 20 October 2021) and identified by its retention indices (relative to n-alkanes C8-C22), mass spectrum matching to authentic standards (when available), and Wiley spectral library collection and NIST library database.

### 4.7. Isolation and Purification of Compounds

n-Hexane fraction (10 g) was fractionated by normal vacuum liquid chromatography (VLC) using column 6 × 30 cm, 50 g. Gradient elution was applied using n-hex.:EtOAC mixtures. The collected fractions (100 mL each) were concentrated and monitored by TLC using the system n-hex.:EtOAC (8:2) and visualized by PAA. Similar fractions were grouped and concentrated to provide three sub-fractions (I1–I3). Subfraction II1 (3.0 g) was further fractionated by column chromatography on silica gel 60 (100 × 1 cm, 50 g), which was eluted as before to afford compound **1** (20 mg), and compound **2** (10 mg), while subfraction II2 (100 mg) resulted in compound **3** (50 mg), and compound **4** (30 mg). Finally, Subfractions II3 (70 mg) was also further fractionated to produce compound **5** (50 mg) and compound **6** (30 mg).

### 4.8. In Vitro Cyclooxygenases Inhibitory Activity

The in vitro inhibitory assays of the isolated compounds against COX-1 and COX-2 were determined by using fluorometric-based screening kits (Biovision, Switzerland) according to the manufacturer’s protocol [46,47,48]. These assays are based on the detection of the florescence produced by prostaglandin G2, i.e., the intermediate product produced by the COX-1 and -2 enzymes. The enzymes solutions were prepared by adding 110 mL of dd. H_2_O to the lyophilized enzymes in the kit. The diluted COX cofactor was formulated by mixing the COX assay buffer (398 mL) and COX Cofactor (2 mL). 5 mL of arachidonic acid were added to 5 mL of NaOH and then diluted by 90 mL of double distilled H_2_O to produce dilute arachidonic acid/NaOH solution. Subsequently, all these prepared solutions were mixed to produce the reaction mixture (80 mL). Different concentrations of the test compounds were added to the previous solution. The reaction mixtures were then incubated at 25 °C for 10 min. The produced florescence (Ex/Em = 535/587 nm) was measured by Tecan Spark microplate reader (Tecan Instruments, Inc., Morrisville, NC, USA). These assays of test compounds, blank, and reference inhibitors were carried out in triplicates. IC_50_ values were calculated by GraphPad software (version 7.0), where the percentage inhibitions were plotted versus the log concentrations. Dividing the IC_50_ calculated for COX-2 by the IC_50_ calculated for COX-2 was applied to determine the Selectivity index (SI).

### 4.9. Molecular Modeling

#### 4.9.1. Docking Analysis

Molecular docking experiments were performed using AutoDock Vina software v. 1.2.0 (Scripps Research Institute, La Jolla, CA, USA) [24,49]. COX-1 and COX-2 crystal structures with PDB codes of 3KK6 and 3HS5 were used for docking experiments. The enzyme’s active site used for docking were located according to the co-crystalized ligands, i.e., celecoxib and arachidonic acid, respectively [50,51], where we set the docking’s grid box to enclose the part of the enzyme that was complexed with this co-crystalized ligand. The ligand-to-binding site shape matching root means square (RMSD) cutoff was set to 2.0 Å. All the docked compounds were energy-minimized inside the determined binding sites. The generated binding poses were visualized by using PyMol software v. 2.4 (http://pymol.org/, (accessed on 20 October 2021), Schrödinger, Mannheim, Germany) [52].

#### 4.9.2. Molecular Dynamic Simulation

The Nanoscale Molecular Dynamics (NAMD) 2.6 software was used for molecular dynamic simulations (MDS) of the generated ligand-enzyme complexes [52], applying the CHARMM27 force field [53]. Briefly, hydrogen atoms were added to the protein structures using the psfgen plugin included in the software of Visual Molecular Dynamic (VMD) v. 1.9.3 (University of Illinois at Urbana–Champaign, IL, USA) [54]. Afterward, the whole generated systems were solvated using water molecules (TIP3P) and 0.15 M NaCl. The MDS output was sampled every 0.1 ns to calculate the root mean square deviation (RMSD). The parameters of compound **4** were justified using the online software the VMD Force Field Toolkit (ffTK) [54]. The binding free energies (ΔG) were calculated using the free energy perturbation (FEP) method and the web-based software CHARMM-GUI Absolute Ligand Binder (http://www.charmm-gui.org/input/gbinding (accessed on 20 October 2021)) [55], was used to generate the input files for NAMD software v. 2.11 (University of Illinois and Beckman Institute, Urbana, IL, USA) required for ΔGs calculations.

### 4.10. In Vitro Antioxidant Activity

#### 4.10.1. Hydrogen Peroxide Scavenging Activity

The residual H_2_O_2_ was determined colorimetrically [56]. Briefly, 20 µL of the sample was added to 500 µL of H_2_O_2_ and incubated for 10 min., at 37 °C. Shortly, 500 µL of enzyme/3,5-dichloro-2-hydroxyl-benzensulfonate mixture was added and incubated at 37 °C for 5 min. The optical density of the colored product was measured at 510 nm using ascorbic acid as a positive control. The percentage of H_2_O_2_ scavenging activity was calculated by using the following formula (Equation (1)).

(1) $$\mathrm{Scavenging}\;\mathrm{activity}=\frac{A\;\mathrm{control}\;–\;A\;\mathrm{sample}}{A\;\mathrm{control}}\times 100$$

IC_50_ of each sample was determined at four different concentrations using Graph pad prism 7 software.

#### 4.10.2. Superoxide Radical Scavenging Activity

The superoxide anion scavenging activity was measured as previously performed by Sreenivasan et al., 2007 [57]. The superoxide anion radicals were produced in Tris–HCL buffer (16 mM, pH 8.0), containing 90 µL of NBT (0.3 mM), 90 µL NADH (0.936 mM) solution, 0.1 mL oil of different concentration (125, 250, 500, and 1000 μg/mL), and 0.8 mL Tris–HCl buffer (16 mM, pH 8.0). The reaction was initiated by adding 0.1 mL PMS solution (0.12 mM) to the mixture, incubated at 25 °C for 5 min., and the optical density was measured at 560 nm, compared to the reference ascorbic acid. The percentage inhibition was calculated using the following formula, Equation (2).

(2) $$\mathrm{Superoxide}\;\mathrm{scavenging}\;\mathrm{activity}=\frac{A\;\mathrm{control}\;–\;A\;\mathrm{sample}}{A\;\mathrm{control}}\times 100$$

IC_50_ was calculated using Graph pad prism 7 software by performing the test at four different concentrations.

### 4.11. Wound Healing Activity

#### 4.11.1. Animal Treatment

Eighteen healthy adult male New Zealand Dutch strain albino rabbits weighing 2.0–2.5 kg was included in the current study. Rabbits were accommodated in a well-ventilated animal house made of polypropylene cages, with a free access to the standard pellet diet and water ad libitum during the whole time of the experiment. In addition, seven days prior to the start of the study, the animals were acclimatized to the standard laboratory conditions, temperature (25 ± 2 °C), relative humidity (44–56%), and light and dark cycles (12:12 h).

#### 4.11.2. Preparation of the Test Samples for the Bioassay

Models of excision wound were used to evaluate the wound healing aptitude of the investigated oil. For the wound models, 0.5 mL of Peters’ elephant-nose fish oil was applied topically on each wounded site immediately after wounds were created.

#### 4.11.3. Circular Excision Wound Model and Experimental Design

Wound was induced in rabbits according to Tramontina et al., 2002 [58]. Briefly, animals were initially anaesthetized by 0.01 mL Ketalar^®^, then the of rabbits’ back hair were shaved carefully. The wounds were created with a 500 mm biopsy punch in a circular form by excising only the skin of the dorsal interscapular region. The wounds were cleaned afterwards with sterile cotton wipe dipped in normal saline and treated according to the experiment. Wounds were kept without any dressings throughout the experimental duration.

The 18 adult male New Zealand Dutch strain albino rabbits were randomly divided into three groups each containing six rabbits: group 1: untreated rabbits (positive control); group 2: rabbits treated topically with Peters’ elephant-nose fish oil (*n*-hexane fraction), twice daily for fourteen days till the wounds has mostly healed; group 3: rabbits treated topically with Mebo^®^ (market comparable) twice daily for fourteen days till the wounds has mostly healed. The experimental study of wound healing assessment was performed twice daily.

#### 4.11.4. Collection of Tissue Samples

A full thickness skin biopsy of entire ulcers from all groups was collected under anesthesia on days 7 and 14. Tissue samples were dissected into two sections. The first part of dissected wound tissue for analysis of gene expression, while the second one was preserved in formalin for the histological study.

#### 4.11.5. Percentage Wound Closure Rate

The dynamic changes in wound area were checked regularly by a camera (Fuji, S20 Pro, Japan) every three days till the wound was completely healed. The wound area was analyzed by Image J 1.49v software (National Institutes of Health, Bethesda, MD, USA) and the wound closure rate was expressed following Equation (3).

(3) $$\mathrm{Wound}\;\mathrm{closure}\;\left(\%\right)=\frac{\mathrm{Area}\;\mathrm{of}\;\mathrm{wound}\;\mathrm{on}\;\mathrm{day}\;0\;–\;\mathrm{Area}\;\mathrm{of}\;\mathrm{wound}\;\mathrm{on}\;\mathrm{day}\;\mathrm{nth}}{\mathrm{Area}\;\mathrm{of}\;\mathrm{wound}\;\mathrm{on}\;\mathrm{day}\;0}\times 100.\;$$

where n represented the number of days, i.e., 3rd, 7th, 10th, and 14th.

#### 4.11.6. Histological Study 

Histological assessment of epidermal & dermal regeneration and collagen deposition and fibroblast predominance were performed. Specimens were taken from the skin on the seventh and the fourteenth days and processed to obtain paraffin blocks containing the tissue. Serial sections about 5–6 µm thick were cut from the paraffin blocks and mounted on glass slides, stained with H&E to be viewed by the light microscope. 

#### 4.11.7. Gene Expression Analysis

Total Ribonucleic acid (RNA) was extracted from skin tissues using the Trizol reagent (Invitrogen, Waltham, MA, USA) following the instructions provided by the manufacturer [59]. The extracted RNA was measured spectrophotometrically on NanoDrop 1000 (Thermo Scientific, Waltham, MA, USA). Complementary Deoxyribonucleic acid (cDNA) was reverse-transcript using 1 µg total RNA by high-capacity reverse transcription kit (Thermo Scientific, USA) with oligo-dT primers. Transcript levels were measured by real-time Polymerase chain reaction (PCR) using the sequence-specific primers, shown in Table 3. Amplification was performed in a step one real time PCR thermal cycler (Thermo Fischer Scientific, Waltham, MA, USA) using the SYBR Green PCR Master Mix (Thermo scientific, USA) following the manufacturer’s instructions. Gene expression levels were determined after normalization to glyceraldehyde 3-phosphate dehydrogenase (GAPDH) as a housekeeping gene. Comparative CT method was applied for estimation of relative gene expression.

### 4.12. Statistical Analysis

Data were expressed as mean ± standard error of the mean (S.E.M) of five replicas (n = 5). Additionally, one-way analysis of variance (ANOVA) followed by Dunnett’s test was applied for all the experiments, where Graph Pad Prism 7 was used for statistical calculations (Graph pad Software, San Diego, CA, USA). Results were considered significant at *p* < 0.05.

## 5. Conclusions 

Peters’ elephant-nose fish oil demonstrated a highly promising wound healing effect in excisional wound model. It accelerated and improved several involved features, including wound contraction, modulation of inflammatory markers, and epithelialization. Moreover, the antioxidant and anti-inflammatory activity of the fish oil contributed to the wound healing activity. For the first time, the results have established fish oil as an alternative approach excisional wound. Nevertheless, further investigation is warranted to clarify the role(s) of fish oil on wound healing in human beings and to test the stability of its constituents to ensure an efficacious formulation of products suitable for wound healing.

## Figures and Tables

**Figure 1:**
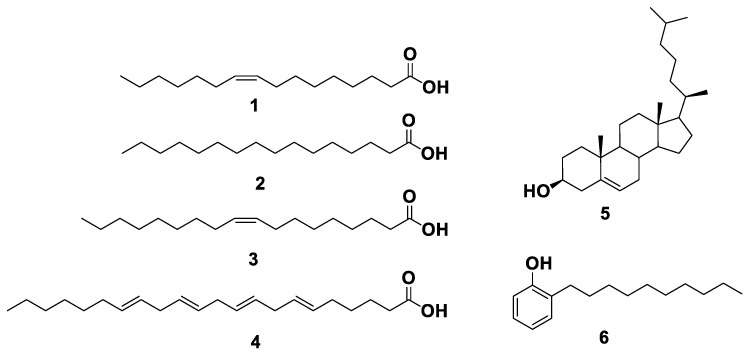
Structures of compounds isolated from Peters’ elephant-nose fish oil.

**Table 1:** Peters’ elephant-nose fish oil composition using GC-MS analysis.

No.	Identified Compound	C:D	Type	Area %	RT	RI
**1**	Caprylic acid	C8:0	SFA	1.18	9.69	892
**2**	Pelargonic acid	C9:0	SFA	0.44	9.39	875
**3**	Caproic acid	C10:0	SFA	0.22	10.05	845
**4**	Lauric acid	C12:0	SFA	1.53	11.18	882
**5**	Myristic acid	C14:0	SFA	2.80	12.92	907
**6**	13-Methyl, Myristic acid	C15:0	SFA	3.20	14.03	909
**7**	Pentadecanoic acid	C15:0	SFA	3.26	15.11	891
**8**	Palmitic acid	C16:0	SFA	4.11 *	15.70	810
**9**	6,9,12-Octadecatrienoic acid	C18:3 (6,9,12)	PUFA	1.67	18.69	841
**10**	7,10,13-Eicosatrienoic acid	C20:3 (7,10,13)	PUFA	1.57	19.57	843
**11**	Arachidonic acid	C20:4 (5,8,11,14)	PUFA	2.45	20.60	931
**12**	6,9,12,15-Docosatetraenoic acid	C22:4 (6,9,12,15)	PUFA	4.63 *	21.59	816
**13**	Eicosa-5,8,11,14,17-pentaenoic acid	C20:5 (5,8,11,14,15)	PUFA	2.65	22.07	878
**14**	7,10-Octadecadienoic acid	C18:2 (7,10)	PUFA	0.75	24.35	882
**15**	10-Heptadecen-8-ynoic acid	C17:2 (8,10)	PUFA	0.45	25.75	678
**16**	9,12-Octadecadienoic acid	C18:2 (9,12)	PUFA	1.91	26.5	871
**17**	4,7,10,13,16,19-Docosahexaenoic acid	C22:6 (4,7,10,13,16,19)	PUFA	5.55 *	30.10	913
**18**	7,10,13,16,19-Docosapentaenoic acid	C22:5 (7,10,13,16,19)	PUFA	0.45	31.31	855
**19**	Palmitoleic acid	C16:1 (7)	MUFA	3.40	28.68	904
**20**	Palmitoleic acid, 15-methyl-	C17:1 (7)	MUFA	1.02	30.54	907
**21**	Margaric acid	C17:0	SFA	3.47	31.23	908
**22**	10-Heptadecenoic acid	C17:1 (10)	MUFA	1.04	32.41	830
**23**	Stearic acid	C18:0	SFA	2.06	33.20	914
**24**	Vaccenic acid	C18:1 (7)	MUFA	1.71	32.01	910
**25**	Oleic acid	C18:1 (9)	MUFA	4.99*	32.51	925
**26**	Nonadecanoic acid	C19:0	SFA	0.67	33.02	815
**27**	Arachidic acid	C20:0	SFA	1.47	36.46	904
**28**	Behenic acid	C22:0	SFA	0.92	39.86	899
**29**	Cholesterol	C27:1 (5)	Sterol	3.99	34.38	913
**30**	Cholestan-3-ol, 2-methylene-, (3α,5α)-	C28:1 (5)	Sterol	0.16	35.38	797
**31**	Heptacosane	C27:0	SHC	3.75	37.61	877
**32**	Dotriacontane	C32:0	SHC	1.68	43.35	851
**33**	Cholesterol margarate	C44:1 (5)	Sterol	0.30	43.83	812
**34**	Cholesta-4,6-dien-3-ol, (3α)-	C27:1 (5)	Sterol	0.51	43.92	890
**35**	Cholesta-3,5-diene	C27:2 (3,5)	Sterol	1.15	44.08	889
**SFA**				25.33%		
**MUFA**				12.16%		
**PUFA**				22.08%		
**SHC**				5.43%		
**Sterol**				6.11 %		
**Total**				71.11%		

RI: retention index relative to n-alkanes, RT: retention time (min), C:D: carbon number to double bond number involving their position, *: major compound, SFA: saturated fatty acid, MUFA: mono-unsaturated fatty acid, PUFA: poly unsaturated fatty acid, SHC: saturated hydrocarbon, %: percentage.

**Table 2:** Inhibitory activity of Peters’ elephant-nose fish oil, and its isolates against both COX-1 and COX-2 calculated as IC_50_ (µM) ± SEM (n = 3).

Compound	COX-2 (µM)	COX-1 (µM)	COX-2/COX-1
**1**	>100	>100	-
**2**	>100	>100	-
**3**	>100	>100	-
**4**	2.41 ± 0.2 *	18.5 ± 0.4 *	0.13
**5**	>100	>100	-
**6**	>100	>100	-
**Crude oil**	15.27 ± 0.3 *	46.33 ± 0.2 *	0.33
**Celecoxib** **^®^**	0.125 ± 0.2 *	2.53 ± 0.3 *	0.05

* Statistically significant at *p* < 0.05.

**Table 3:** Primers used for real time PCR experiments.

Gene Name	GenBank Accession		
IL-β_1_	NC_013670.1	Forward	5′-AGCTTCTCCAGAGCCACAAC-3′
		Reverse	5′-CCTGACTACCCTCACGCACC-3′
GAPDH	NC_013676.1	Forward	5′-GTCAAGGCTGAGAACGGGAA-3′
		Reverse	5′-ACAAGAGAGTTGGCTGGGTG-3′
TGF-β_1_	NC_013672.1	Forward	5′-GACTGTGCGTTTTGGGTTCC-3′
		Reverse	5′-CCTGGGCTCCTCCTAGAGTT-3′
TNF-α	NC_013680.1	Forward	5′-GAGAACCCCACGGCTAGATG-3′
		Reverse	5′-TTCTCCAACTGGAAGACGCC-3′

## Supplementary Materials

The following are available online at https://www.mdpi.com/article/10.3390/md19110605/s1, Figure S1: GC-MS spectrum for Peters’ elephant-nose fish oil; Figures S2–S13: 1D and 2D NMR spectra of compounds **1**–**6**.
