# Supplementary material for: Wound Healing Metabolites from Peters’ Elephant-Nose Fish Oil: An In Vivo Investigation Supported by In Vitro and In Silico Studies
Source: Mar Drugs. 2021 Oct 26;19(11):605. doi: 10.3390/md19110605 (PMC8625051; doi:10.3390/md19110605)
Supplement: Supplementary file 1 [file marinedrugs-19-00605-s001.zip › marinedrugs-1416811-supplementary.pdf]

SUPPLEMENTARY DATA

Wound Healing Metabolites from Peters' Elephant-Nose Fish Oil: an *in vivo* Investigation Supported by *in vitro* and *in silico* Studies

Faisal Alsenani, Ahmed M. Ashour, Mubarak A. Alzubaidi, Ahmed F. Azmy, Mona H. Hetta, Dalia H. Abu-Baih, Mahmoud A. Elrehany, Ahmed Zayed*, Ahmed M. Sayed, Usama Ramadan Abdelmohsen*, Abeer H. Elmaidomy

List of Contents

Figure S1. GC/MS spectrum for Peters' elephant-nose fish oil

Figure S2. ^{1}H NMR spectrum of compound **1** measured in DMSO-d_6 at 400 MHz

Figure S3. DEPT-Q NMR spectrum of compound **1** measured in DMSO-d_6 at 100 MHz

Figure S4. ^{1}H NMR spectrum of compound **2** measured in DMSO-d_6 at 400 MHz

Figure S5. DEPT-Q NMR spectrum of compound **2** measured in DMSO-d_6 at 100 MHz

Figure S6. ^{1}H NMR spectrum of compound **3** measured in $CDCl_3$ at 400 MHz

Figure S7. DEPT-Q NMR spectrum of compound **3** measured in $CDCl_3$ at 100 MHz

Figure S8. ^{1}H NMR spectrum of compound **4** measured in DMSO-d_6 at 400 MHz

Figure S9. DEPT-Q NMR spectrum of compound **4** measured in DMSO-d_6 at 100 MHz

Figure S10. ^{1}H NMR spectrum of compound **5** measured in $CDCl_3$ at 400 MHz

Figure S11. DEPT-Q NMR spectrum of compound **5** measured in $CDCl_3$ at 100 MHz

Figure S12. ^{1}H NMR spectrum of compound **6** measured in $CDCl_3$ at 400 MHz

Figure S13. DEPT-Q NMR spectrum of compound **6** measured in $CDCl_3$ at 100 MHz

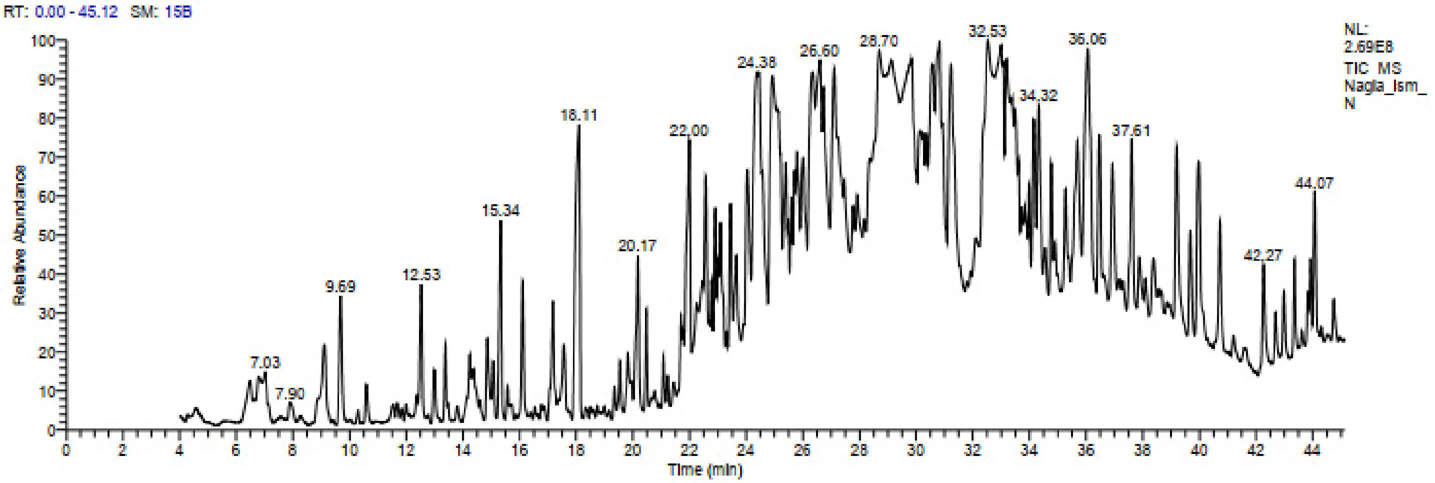

Figure S1. GC/MS spectrum for Peters' elephant-nose fish oil

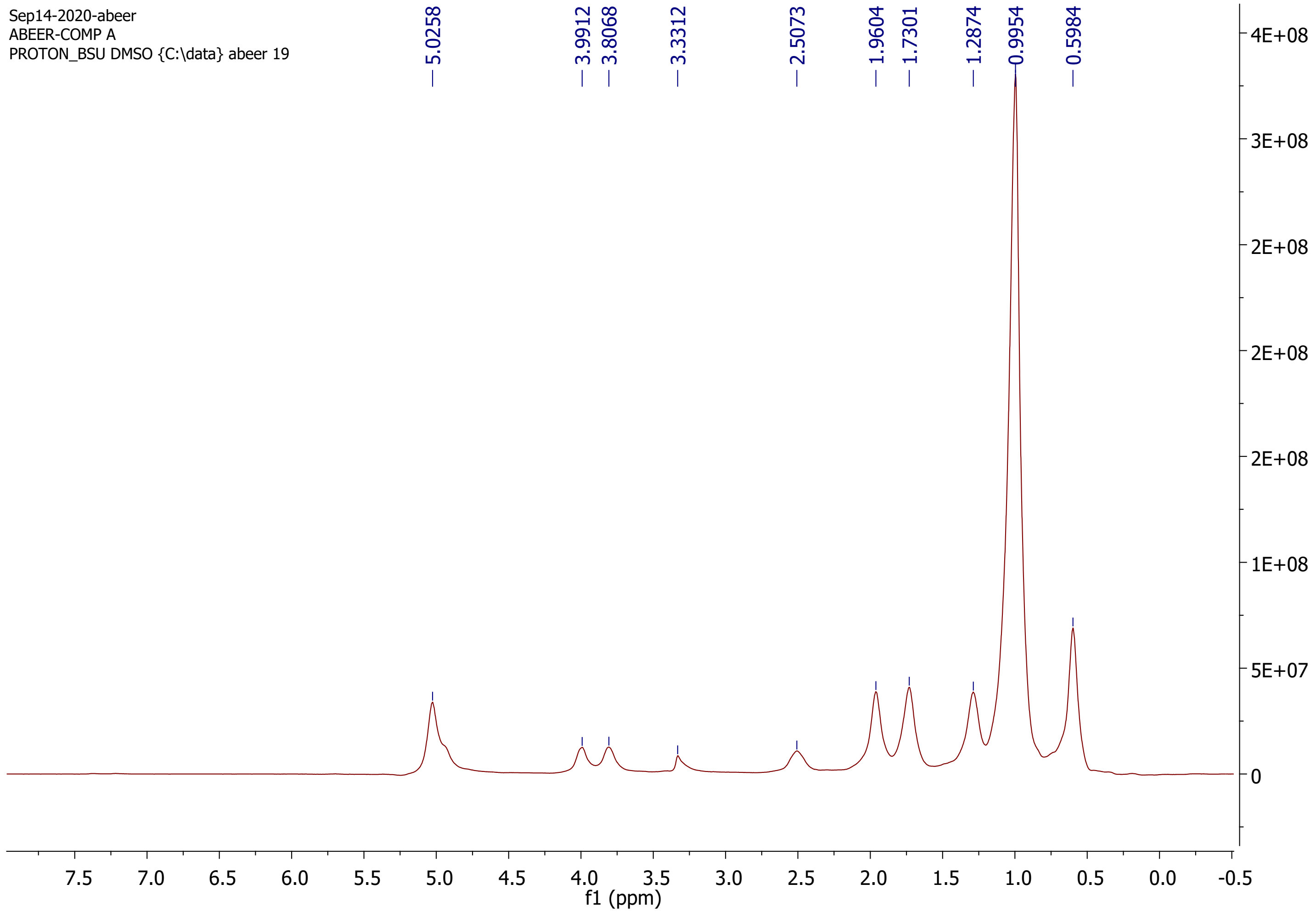

Figure S2. ^{1}H NMR spectrum of compound **1** measured in DMSO-d_6 at 400 MHz

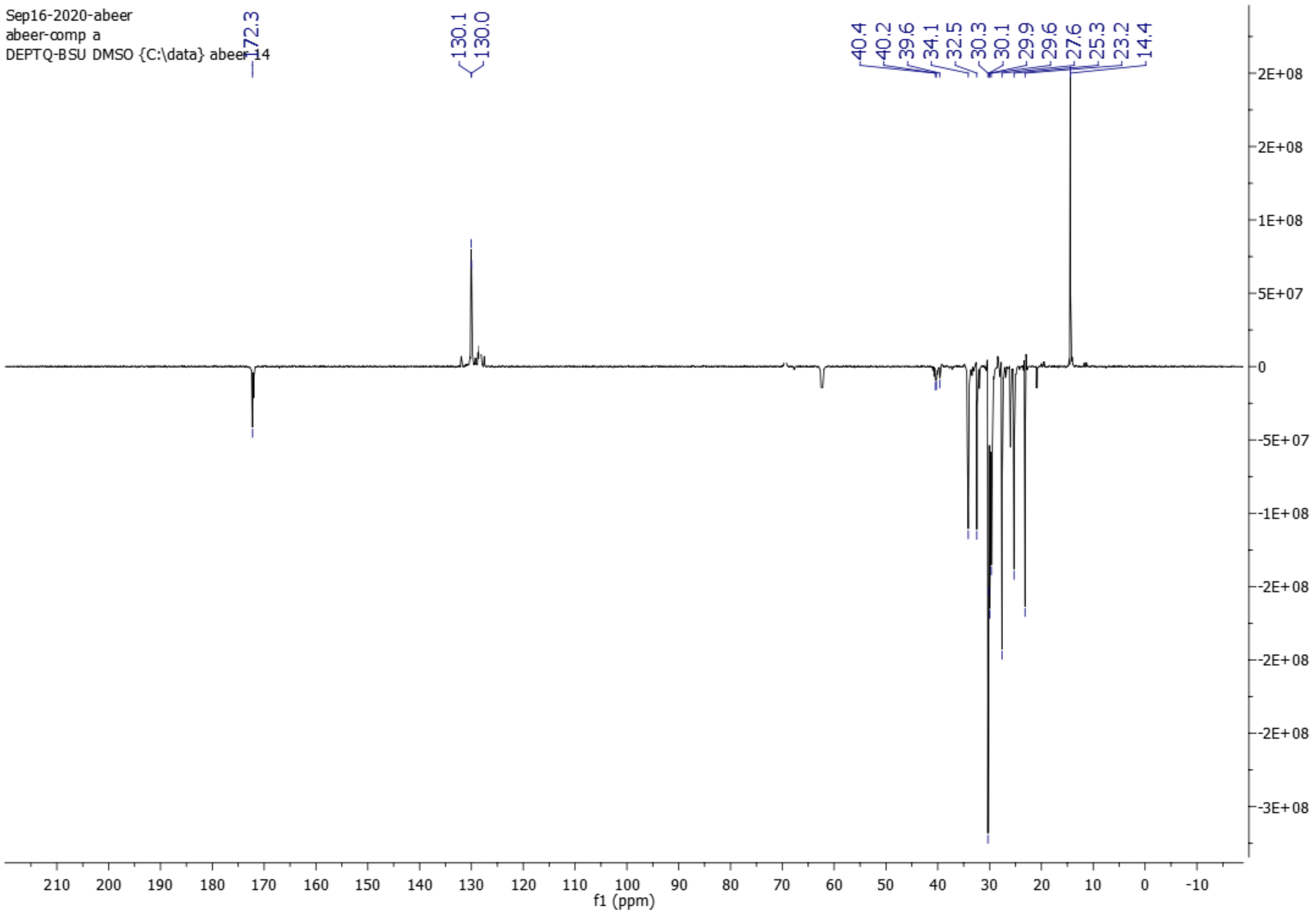

Figure S3. DEPT-Q NMR spectrum of compound **1** measured in DMSO-d_6 at 100 MHz

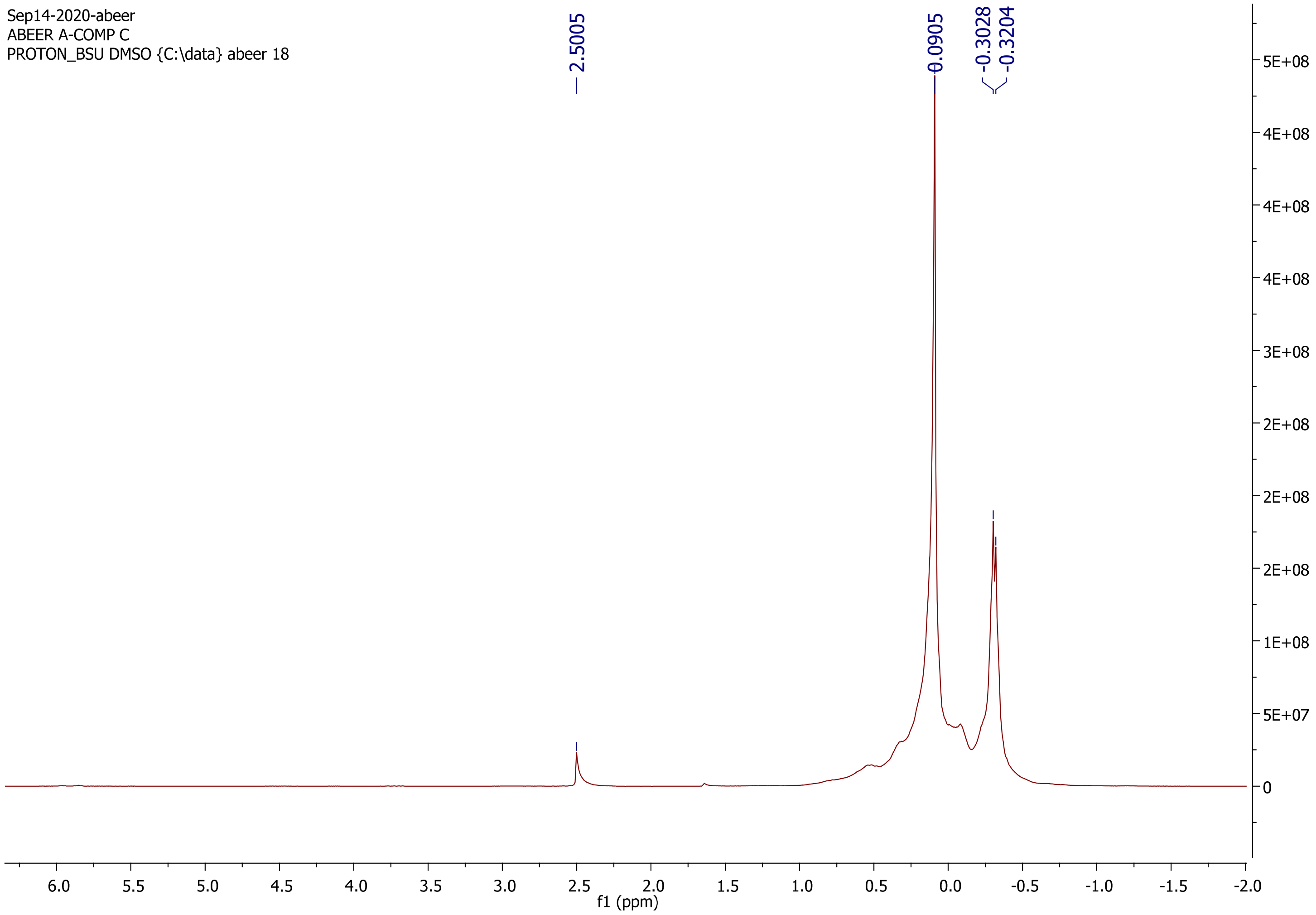

Figure S4. ^{1}H NMR spectrum of compound **2** measured in DMSO-d_6 at 400 MHz

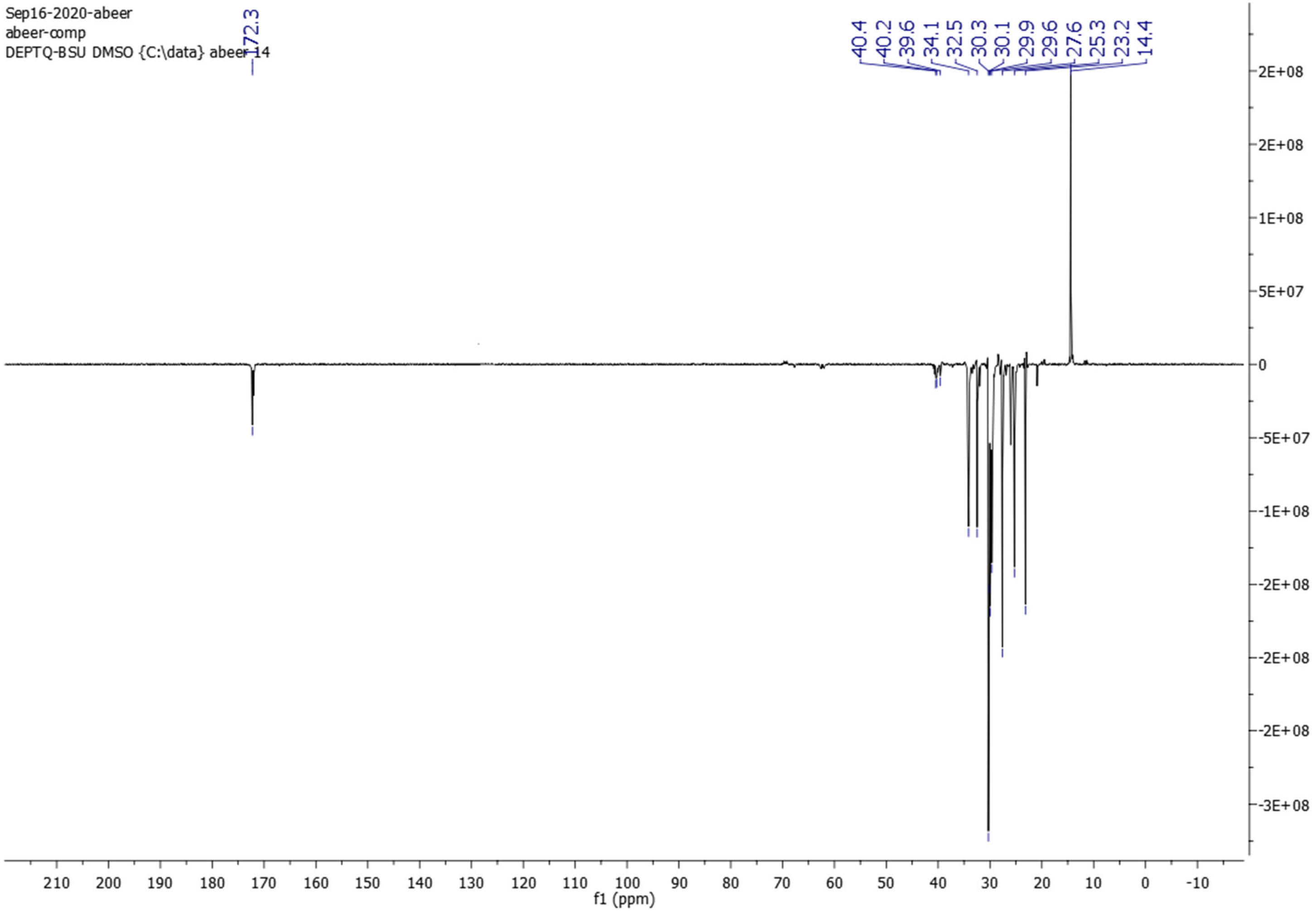

Figure S5. DEPT-Q NMR spectrum of compound **2** measured in DMSO-d_6 at 100 MHz

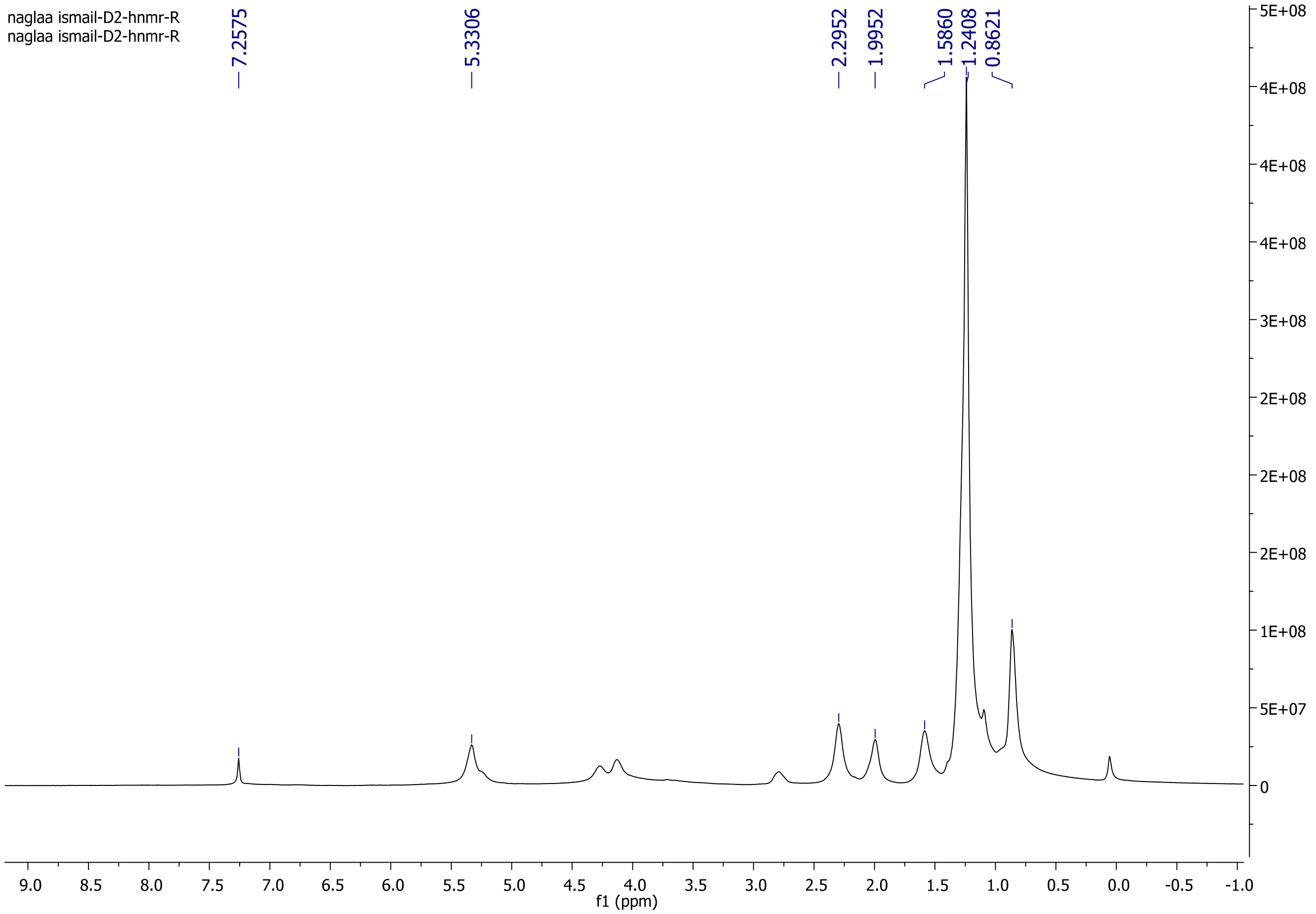

Figure S6. ^{1}H NMR spectrum of compound **3** measured in $CDCl_3$ at 400 MHz

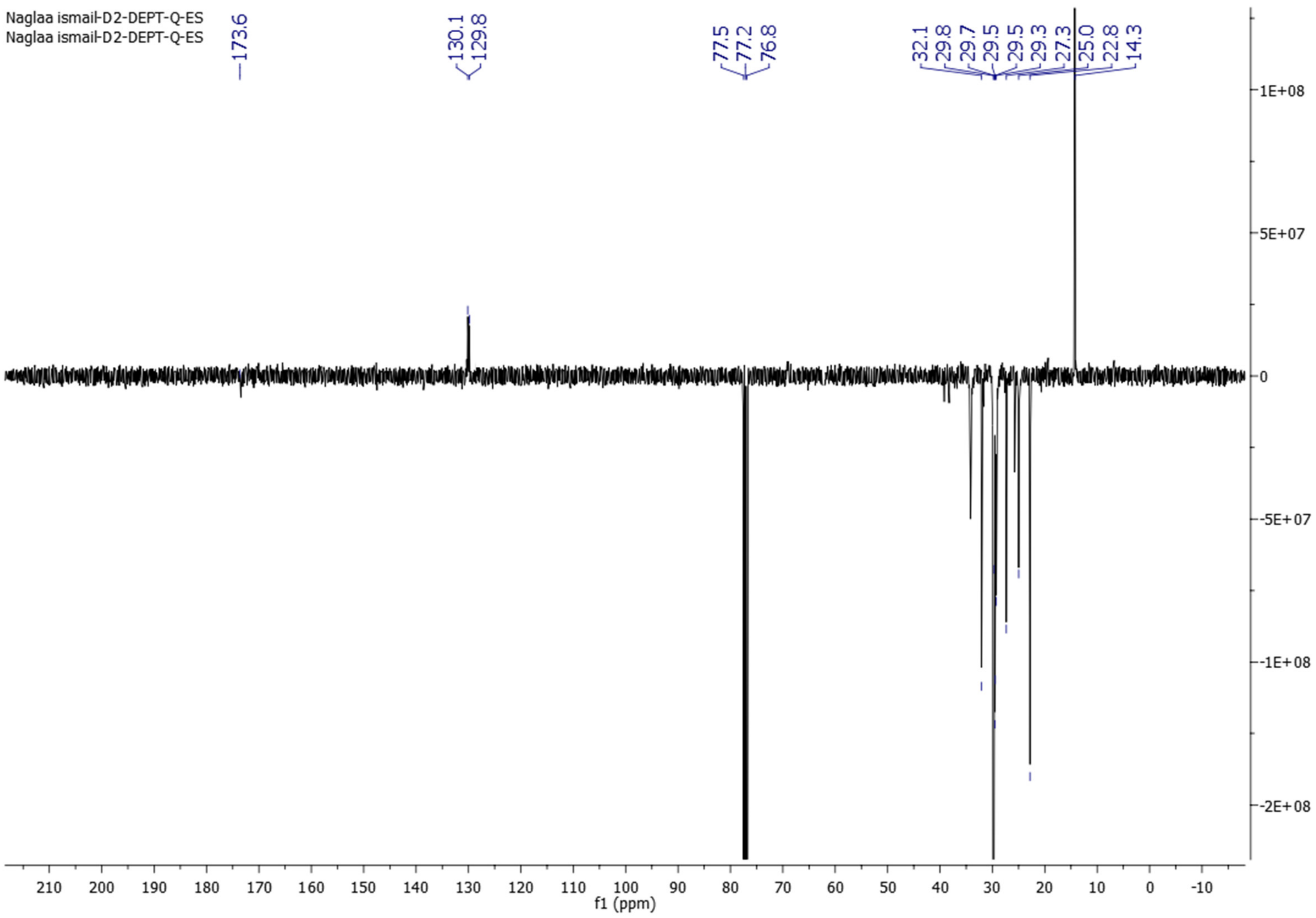

Figure S7. DEPT-Q NMR spectrum of compound **3** measured in $CDCl_3$ at 100 MHz

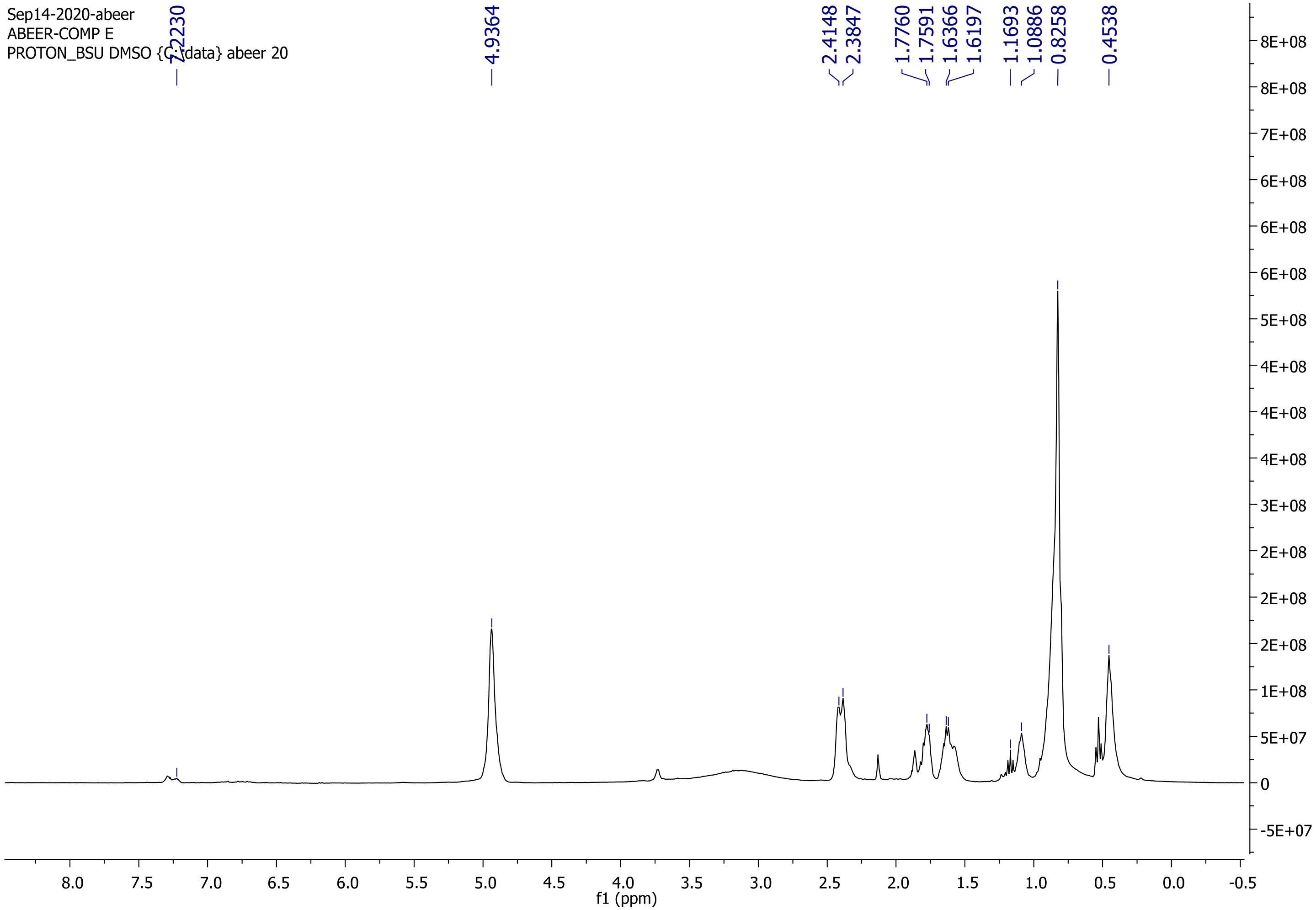

Figure S8. ^{1}H NMR spectrum of compound **4** measured in DMSO-d_6 at 400 MHz

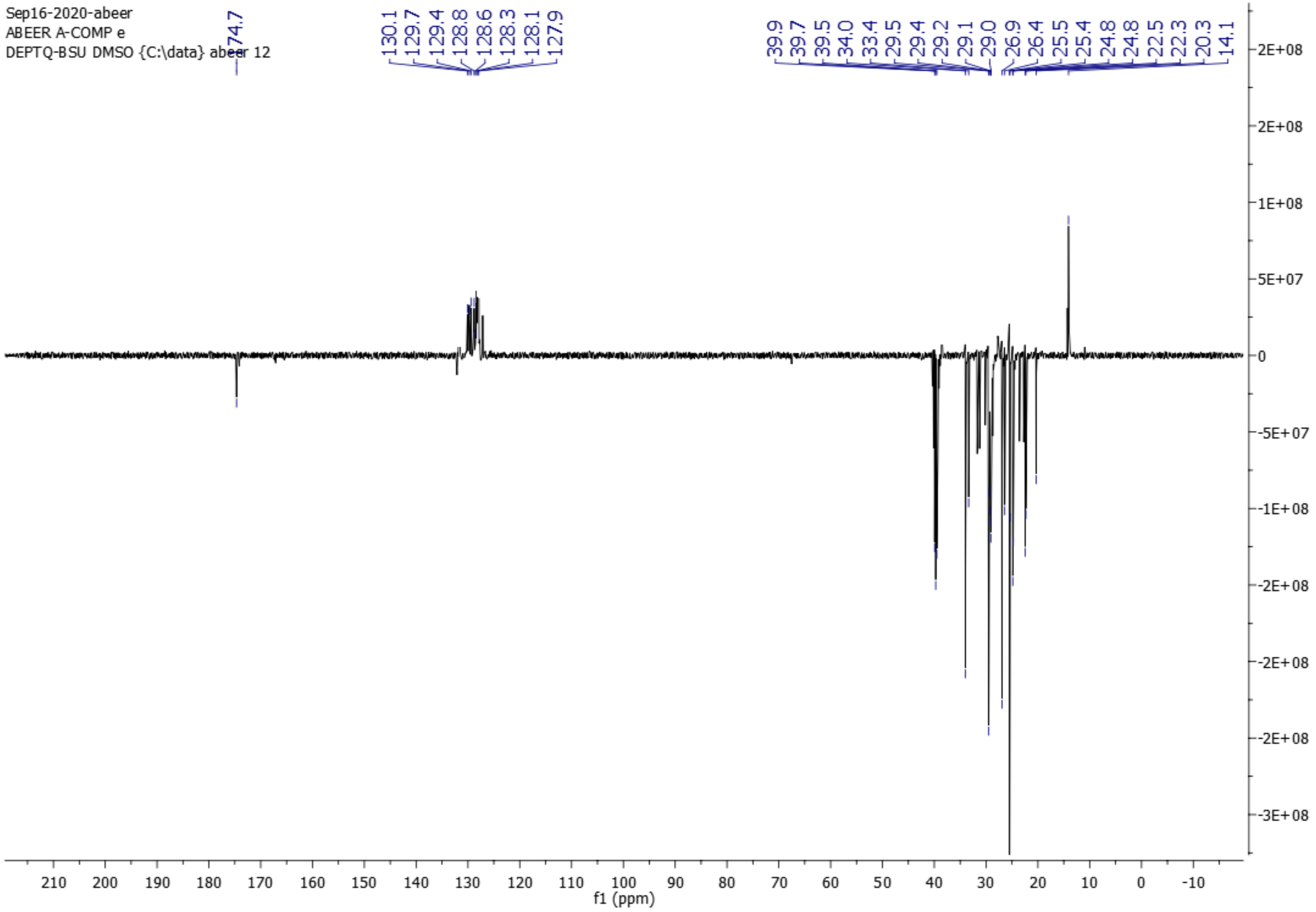

Figure S9. DEPT-Q NMR spectrum of compound **4** measured in DMSO-d_6 at 100 MHz

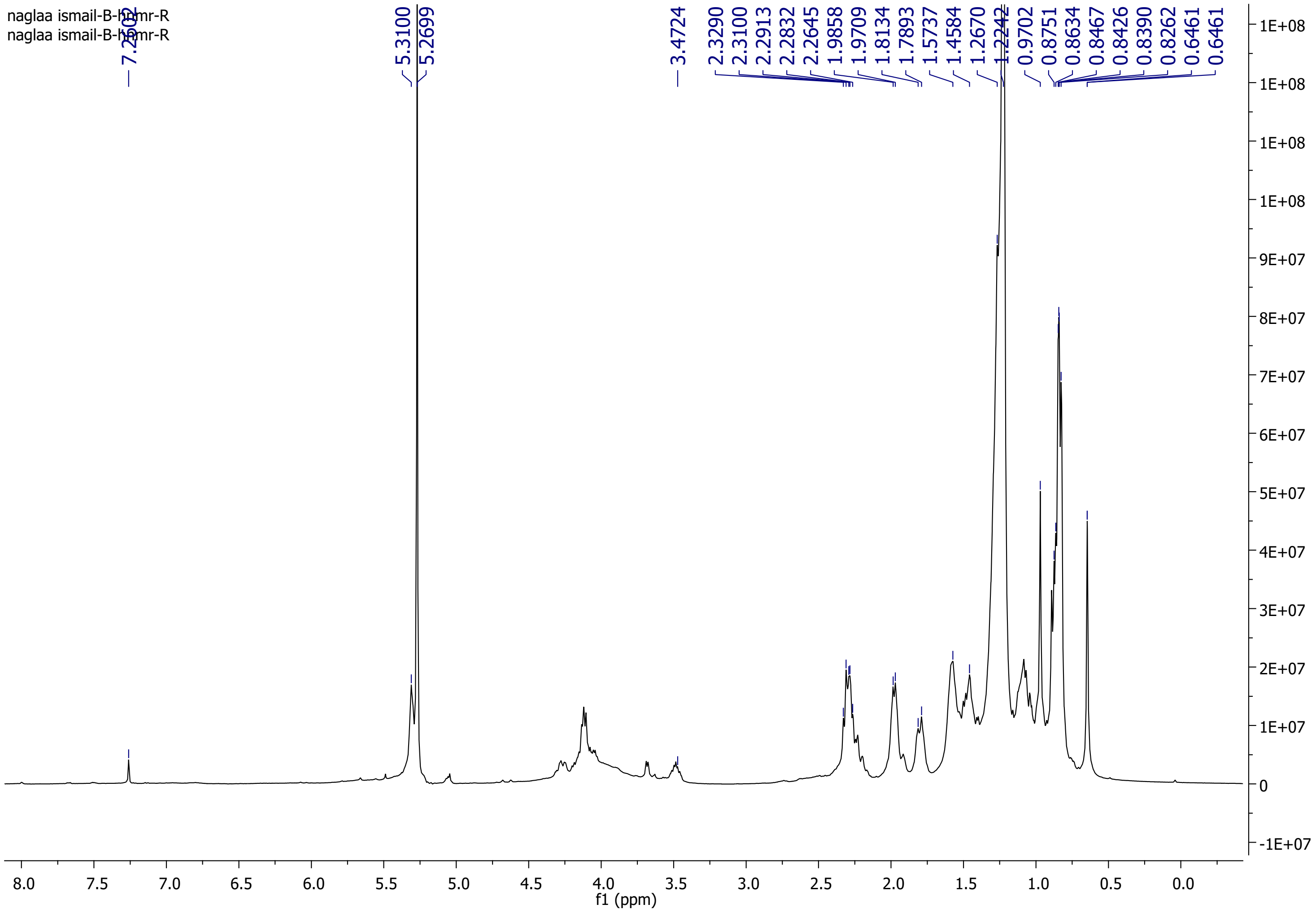

Figure S10. ^1H NMR spectrum of compound **5** measured in $CDCl_3$ at 400 MHz

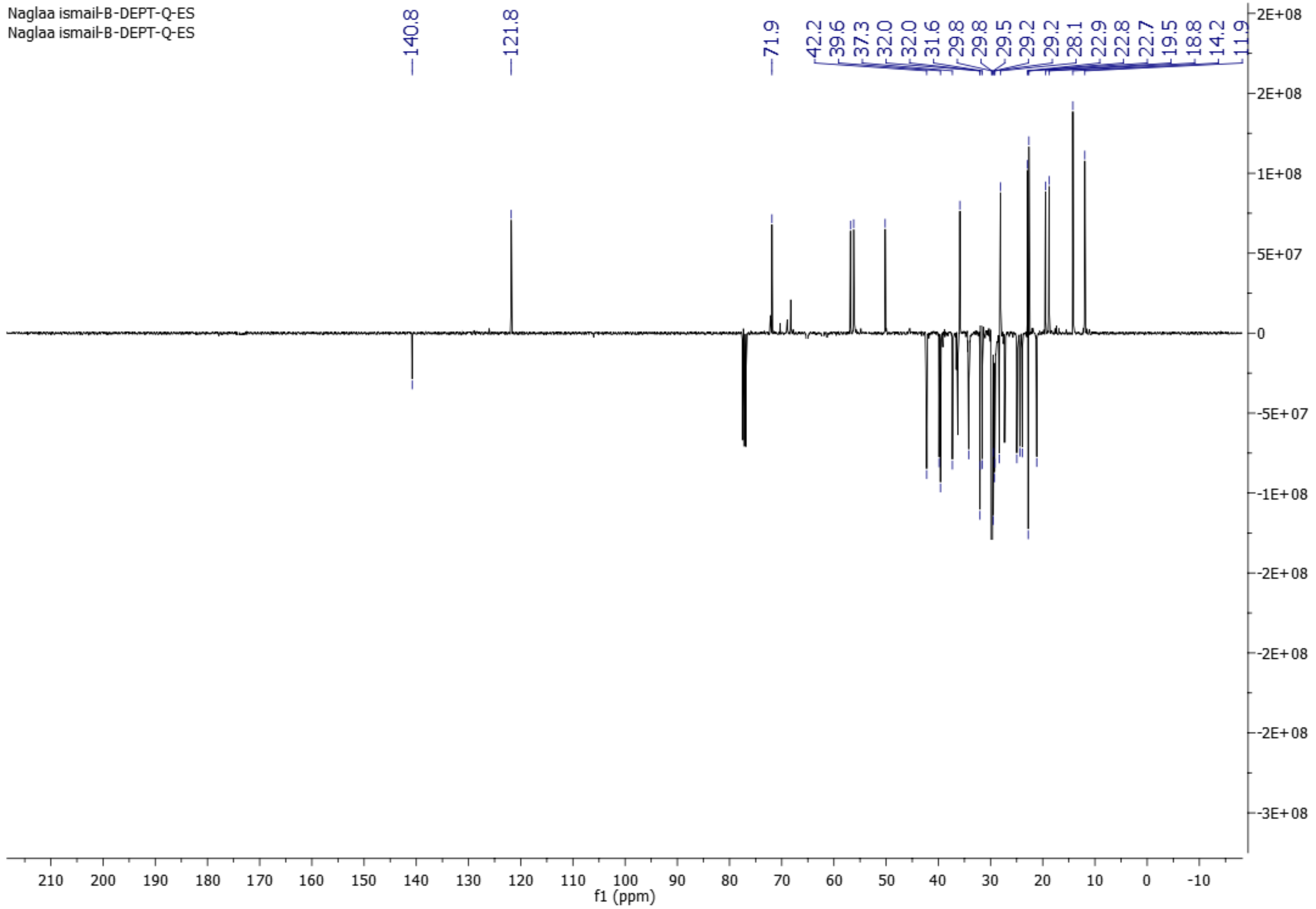

Figure S11. DEPT-Q NMR spectrum of compound **5** measured in $CDCl_3$ at 100 MHz

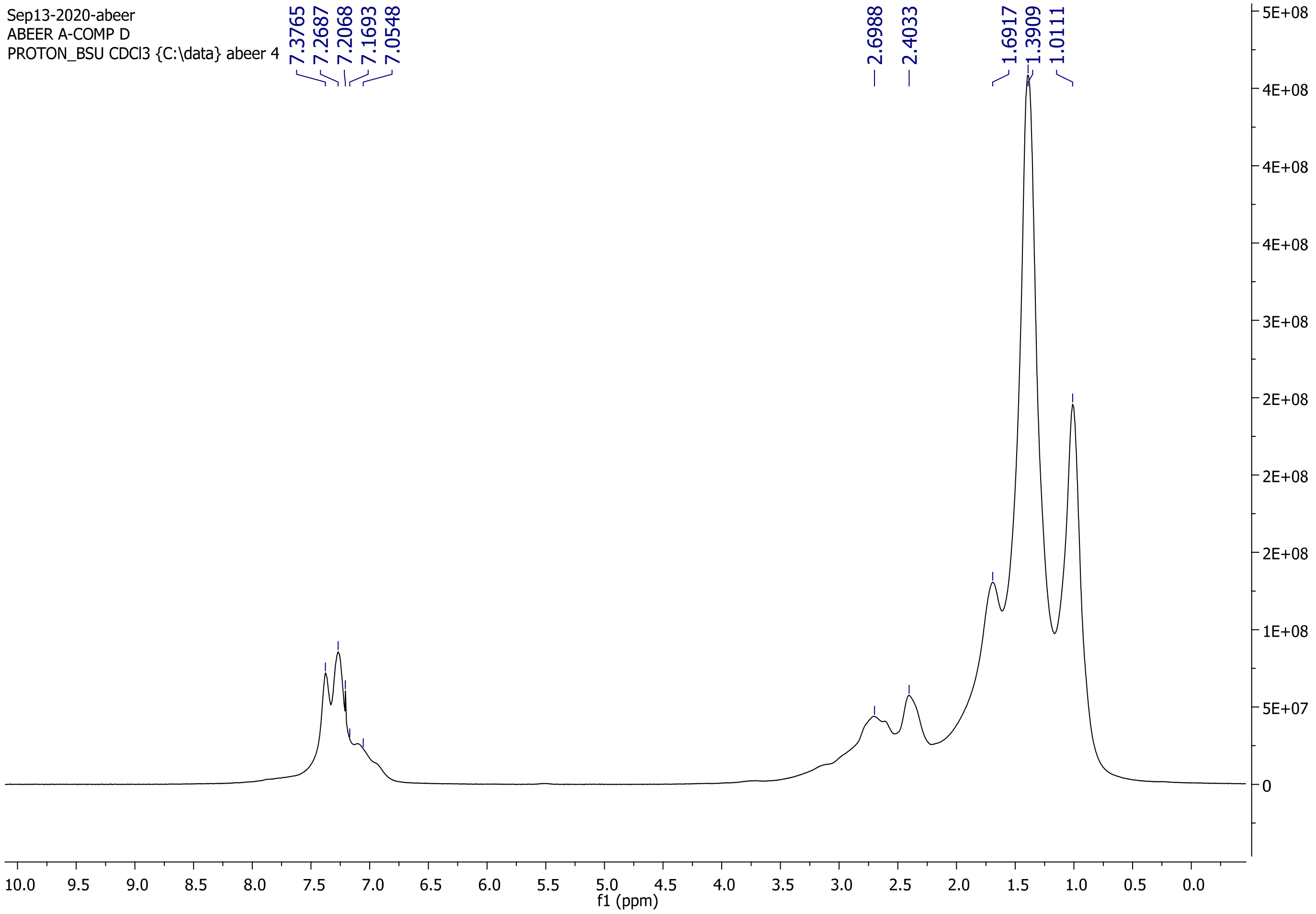

Figure S12. ^{1}H NMR spectrum of compound **6** measured in $CDCl_3$ at 400 MHz

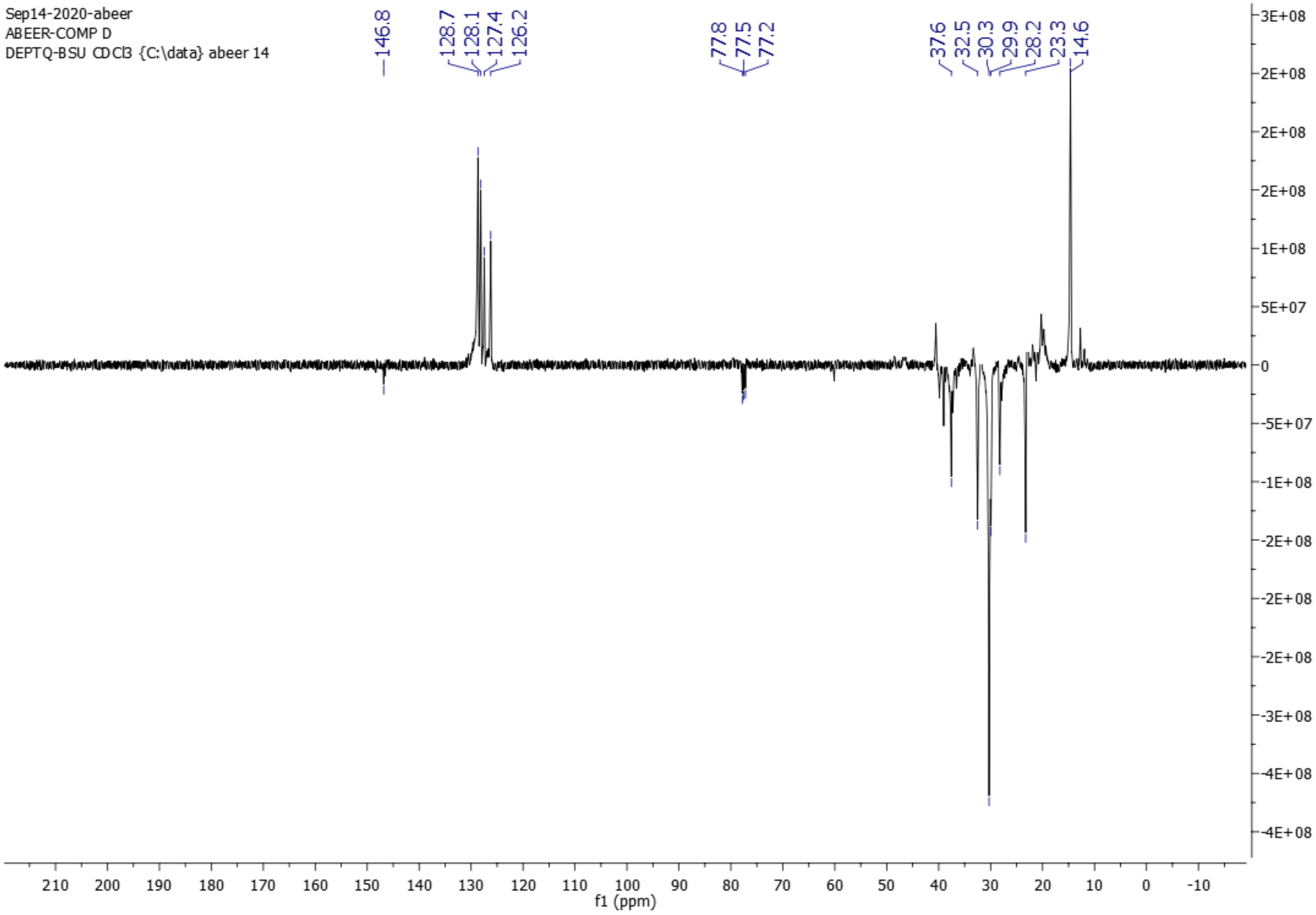

Figure S13. DEPT-Q NMR spectrum of compound **6** measured in $CDCl_3$ at 100 MHz
